# Altered vitamin B12 metabolism in the central nervous system is associated with the modification of ribosomal gene expression: new insights from comparative RNA dataset analysis

**DOI:** 10.1007/s10142-023-00969-6

**Published:** 2023-01-23

**Authors:** Aimee Rachel Mathew, Virve Cavallucci, Marco Fidaleo

**Affiliations:** 1 Department of Biology and Biotechnology Charles Darwin, University of Rome Sapienza, 00185 Rome, Italy; 2 Fondazione Policlinico Universitario A. Gemelli IRCCS, 00168 Rome, Italy; 3 Institute of General Pathology, Università Cattolica del Sacro Cuore, 00168 Rome, Italy; 4 Research Center for Nanotechnology for Engineering of Sapienza (CNIS), University of Rome Sapienza, 00185 Rome, Italy

**Keywords:** Cobalamin, Vitamin B12, Central nervous system, Neuronal homeostasis, Ribosome gene regulation

## Abstract

Recent studies have confirmed the direct role of vitamin B12 (VitB12) in the central nervous system (CNS) homeostasis; nevertheless, the detailed mechanisms are poorly understood. By analyzing RNA-Seq and microarray datasets obtained from databanks, this study aims to identify possible basic mechanisms, related to the brain, involved in altering the gene expression under VitB12 deficiency mimicking conditions. The database inquiry returned datasets generated from distinctly heterogeneous experimental sets and considering the quality and relevance requirements, two datasets from mouse and one from rat models were selected. The analyses of individual datasets highlighted a change in ribosomal gene expression in VitB12 deficiency mimicking conditions within each system. Specifically, a divergent regulation was observed depending on the animal model: mice showed a down regulation of the ribosomal gene expression, while rats an upregulation. Interestingly, *E2f1* was significantly upregulated under VitB12 deficiency mimicking conditions in the animal models, with a greater upregulation in rats. The rat model also revealed putative E2F1 Transcription Factor Binding Sites (TFBSs) in the promoter of the differently regulated genes involved in ribosomal gene expression. This suggested the possibility that E2F1, being greater expressed in rats, could activate the ribosomal genes having E2F1 TFBSs, thus giving a plausible explication to the divergent regulation observed in animal models. Despite the great diversity of the experimental sets used to generate the datasets considered, a common alteration of the ribosomes exists, thereby indicating a possible basic and conserved response to VitB12 deficiency. Moreover, these findings could provide new insights on E2F1 and its association with CNS homeostasis and VitB12 deficiency.

**Graphical Abstract:**

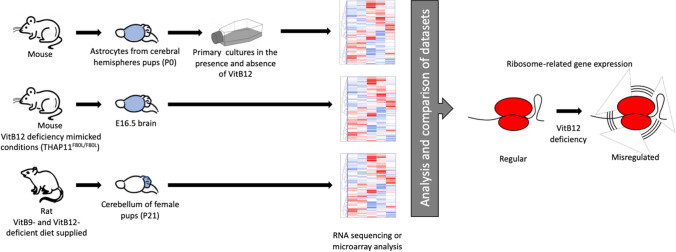

**Supplementary Information:**

The online version contains supplementary material available at 10.1007/s10142-023-00969-6.

## Introduction

Vitamin B12 (VitB12), also known as cobalamin, is an essential micronutrient synthesized exclusively by certain bacteria and archaea, which can enter the food chain of human beings due to their interaction with animals and plants (Salinas et al 2018; Watanabe and Bito 2018). The uptake of VitB12 is a multi-step process and several different alterations can impair its absorption. The estimated reserve of VitB12 in the human liver is roughly about 2–5 mg, in spite of its daily requirement being 1–4 μg. Therefore, the symptoms of VitB12 deficiency develop 3–5 years from the start of malabsorption and then a possible recovery from the deficiency may not be reversible (Henríquez et al 2007). VitB12 is of vital importance due to its significant role as the cofactor in two fundamental biochemical reactions. One such reaction takes place in the mitochondria, where VitB12, in the form of adenosylcobalamin, is responsible for the synthesis of succinyl-CoA from methylmalonyl-CoA and biotin. The other reaction occurs in the cytoplasm, where VitB12, in the form of methylcobalamin, participates in the synthesis of methionine from folic acid (VitB9) and homocysteine (Allen 2012; Kräutler 2012). Therefore, VitB12 deficiency leads to the accumulation of methylmalonic acid and homocysteine, respectively, which are ultimately toxic at high concentrations. VitB12 deficiency can be equally detrimental for both the older population and children (Fidaleo et al. 2021).

VitB12 deficiency can give rise to a broad range of, yet often unrecognized, symptoms (such as weakness, loss of appetite, and weight) and can severely affect human health by impairing fertility, embryo development and progressing into severe disease conditions such as megaloblastic anemia, cancer, and neurological modifications (namely numbness and tingling in limbs, depression, confusion, dementia, and optic neuropathy) (Andrès et al. 2007; Ata et al. 2020; Sangle et al 2020). Furthermore, VitB12 deficiency is associated with cognitive functions and can present as risk factor for Alzheimer’s disease (AD) (Calderón-Ospina and Nava-Mesa 2020). It is also linked to many other neuronal disorders, such as Wernicke’s encephalopathy, depression, subacute combined degeneration of the spinal cord, and peripheral neuropathy (Calderón-Ospina and Nava-Mesa 2020). In children, VitB12 deficiency due to the strict vegan diet of lactating mothers or malnourishment can affect their development and result in movement disorders and developmental delays. The various inborn errors of metabolism, also known as congenital metabolic diseases or inherited metabolic disorders, affecting absorption (intrinsic factor deficiency), transport (transcobalamin deficiency) and intracellular metabolism of VitB12 (combined methylmalonic acidemia and homocystinuria depending on the genes involved) rely on a specific requirement of supplementation/treatment with VitB12 that allow patients to survive and, in some cases, improve mental impairments (Watkins and Rosenblatt 2011). Furthermore, VitB12 deficiency can severely affect myelin formation, remyelination and regeneration of nerves after a peripheral injury and can also lead to the incorporation of abnormal fatty acids into neuronal cells and modify the level of some neurotransmitters (Duncan et al. 2018; Nielsen et al 2012; Nishimoto et al. 2015). Although medical evidence suggests a direct role of VitB12 in the CNS homeostasis, the detailed mechanisms are not yet characterized in depth. Inborn defects linked to VitB12 uptake and metabolism can help in formulating hypotheses, regarding the possible role of a lack of VitB12 in the pathology, that include byproduct toxicity and missing products of the reaction, impaired methylation capacity, alteration in the level of oxidative stress and modification non-directly linked to the enzymatic functions (Huemer et al. 2017).

Medical approaches are devoted to preventing death and severe damage to organs and the scarce knowledge currently available do not allow researchers to develop possible interventions for recovering neurological impairments, which are more common in the case of late diagnosis. Due to the crucial role VitB12 plays, together with its importance in being an essential micronutrient, a conserved and basal mechanism that may involve the gene expression can be hypothesized as a result of VitB12 deficiency or deficiencies in VitB12 metabolism. Therefore, keeping this in mind for the present study, databases were queried to search for RNA-Seq and microarray datasets regarding the CNS and were individually analyzed. The results obtained were then compared in order to highlight the possible common features and the findings reported in this study could provide insights into future research towards medical interventions.

## Methods

### Data acquisition and analyses

To proceed with the RNA gene expression data analyses, the datasets were obtained from the Gene Expression Omnibus (GEO) in the NCBI website (https://www.ncbi.nlm.nih.gov/) and ArrayExpress (https://www.ebi.ac.uk/arrayexpress/).

The RNA-Seq or microarray datasets were obtained from the GEO DataSets archive from the NCBI and ArrayExpress website. In order to obtain the datasets relevant to the role of VitB12 in CNS homeostasis, queries were initiated including the keywords “Cobalamin and brain” or “Vitamin B12 and brain” or “Cobalamin and CNS” or “Vitamin B12 and CNS.” Such queries gave 61 unique search results pertaining to different experiments conducted on different organisms, organs and tissues. From these search results, four datasets were chosen as the CNS models with alterations in the VitB12 metabolism, thereby making them suitable for the present study (Table 1).

**Table 1:** Datasets employed for the analyses

GEO acc. no	Title	Organism	Experimental Data type	Original dataset	Date of publish	References
GSE103417	Inherited disorders of cobalamin metabolism disrupt nucleocytoplasmic transport of mRNA through impaired methylation/phosphorylation of HuR	Mus musculus	Expression profiling by array	1. Undifferentiated WT N1E-115 cells; 2. Undifferentiated N1E-115 cells expressing TO chimeric proteins; 3. Undifferentiated N1E-115 cells expressing OT chimeric proteins; 4. Differentiated WT N1E-115 cells; 5. Differentiated N1E-115 cells expressing TO chimeric proteins; 6. Differentiated N1E-115 cells expressing OT chimeric proteins	Jul 20, 2018	Battaglia-Hsu et al. 2018
GSE99113	RNA-seq for understanding effects of vitamin B12 removal on astrocyte culture	Mus musculus	Expression profiling by high throughput sequencing	1. Serum-free media; 2. Serum-free and VitB12-free media	May 17, 2022	Jonnalagadda et al. 2022
GSE161763	CblX disease is both an inborn error of cobalamin metabolism and a ribosomopathy	Mus musculus	Expression profiling by high throughput sequencing; Genome binding/occupancy profiling by high throughput sequencing	1. WT; 2. THAP11F80L/F80L	Nov 15, 2021	Chern et al. 2022
GSE104164	Wnt-signaling pathways are dysregulated in female cerebellum following an early methyl donor deficiency in a rat nutritional model	Rattus norvegicus	Expression profiling by array	1. Standard diet; 2. VitB9 and VitB12 deficiency, MDD (methyl donor deficiency)	May 11, 2018	Willekens et al. 2019

GSE103417 is an expression profiling by array obtained from the neuroblastoma cells (N1E-115). N1E-115 cells were engineered to express a chimeric protein by fusing oleosin to the N-terminal (TO) or C-terminal (OT) of transcobalamin. TO chimeric proteins had an increased binding affinity towards VitB12, whereas the OT chimeric proteins had no detectable VitB12 binding activity. Thus, the expression of OT chimeric proteins led to the sequestering of substrates and helped in mimicking an appropriate VitB12 deficiency condition. Furthermore, neuroblastoma cells can have a proliferative phenotype (tumor status) or can be differentiated towards a non-proliferative motor-like neuron phenotype (Battaglia-Hsu et al. 2009, 2018). Therefore, the datasets from the non-proliferative motor-like neuron phenotypes were considered for further analyses.

GSE99113 is a high throughput sequencing dataset of the astrocytes obtained from cerebral hemispheres of postnatal day 0 C57BL/6 J pups. In this experiment, VitB12 deficiency was induced by maintaining the astrocyte cultures under serum-free conditions (DMEM/F12) and VitB12-free medium (custom-synthesized medium) (Jonnalagadda et al 2022).

GSE161763 is a high throughput sequencing dataset obtained from the E16.5 brain of a mouse model carrying the mutated protein THAP11^F80L/F80L^. THAP11 is a transcription factor that regulates the transcription of *Mmachc* (a key gene responsible for the intracellular cobalamin metabolism). Here, the change of a phenylalanine to leucine at position 80 of THAP11 (THAP11^F80L/F80L^) determines a decrease in *Mmachc* gene transcription and depletion of MMACHC protein, thereby mimicking the VitB12 deficiency condition (Chern et al. 2022).

GSE104164 is an expression profiling by array obtained from the ground cerebellum of female rat pups under nutritional methyl donor deficiency (MDD). Nutritional MDD was generated by feeding female rats with a diet deficient in folate (VitB9) and VitB12 and lowered in choline, starting from one month before pregnancy until the pups were weaned at postnatal day 21 (P21) (Willekens et al. 2019).

For analyzing the RNA-Seq and microarray datasets, iDEP (integrated differential expression and pathway analysis), a web-based tool, was employed (http://ge-lab.org/idep/) to perform exploratory data analysis (EDA) (Ge et al 2018).

Gene expression matrices were built from TXT files downloaded from GEO DataSets. Datasets were filtered based on the parameters mentioned in Table S1. The quality of datasets was assessed by hierarchical clustering and principal component analysis (PCA). The differentially expressed genes (DEG) were detected by DESeq2 package and the co-expressed genes were used to identify Gene Ontology (GO) using cellular component as a gene set. Also, genes emerging from DEG analysis were used to conduct an enrichment of transcription factor (TF) binding motif in the upstream 600 base pairs (bps) of the promoters of the highlighted genes. The number of replicas for samples of each dataset, false discovery rate (FDR) cutoff, and minimal fold change and specific GO considered are listed in Table S1.

PGSEA (parametric gene set enrichment analysis) package was used to run the PAGE (parametric analysis of gene set enrichment) algorithm, which performs one-sample *t*-test on each gene included in the gene dataset chosen (GO cellular component), to identify the altered pathways.

### ChIP-X enrichment analysis 3

TF enrichment analysis on the genes that were considered to build the “Ribosome” pathway (derived from the Enrichment analysis in the differentially expressed genes (DEG) analysis on mice considering GO cellular component gene set) was performed by ChIP-X enrichment analysis 3 (ChEA3) considering “Literature” dataset (https://amp.pharm.mssm.edu/ChEA3) (Keenan et al. 2019).

### In silico analyses

Sequencing alignment was performed by the Pairwise Sequence Alignment—EMBOSS Needle that uses Needleman-Wunsch algorithm (https://www.ebi.ac.uk/).

Identification of the putative Transcription Factor Binding Sites (TFBSs) in the promoter regions were performed using PROMO (http://alggen.lsi.upc.es/) (Farré et al. 2003; Messeguer et al. 2002). Sequences were related to matrixes using Quandt and co-workers similarity algorithm, and the default threshold for dissimilarity is 15% (Quandt et al 1995). Sequences used for the alignment and identification of the putative TFBSs have been obtained from the NCBI website (https://www.ncbi.nlm.nih.gov/).

## Results

### Pre-processing and exploratory data analysis

**Fig. 1:**
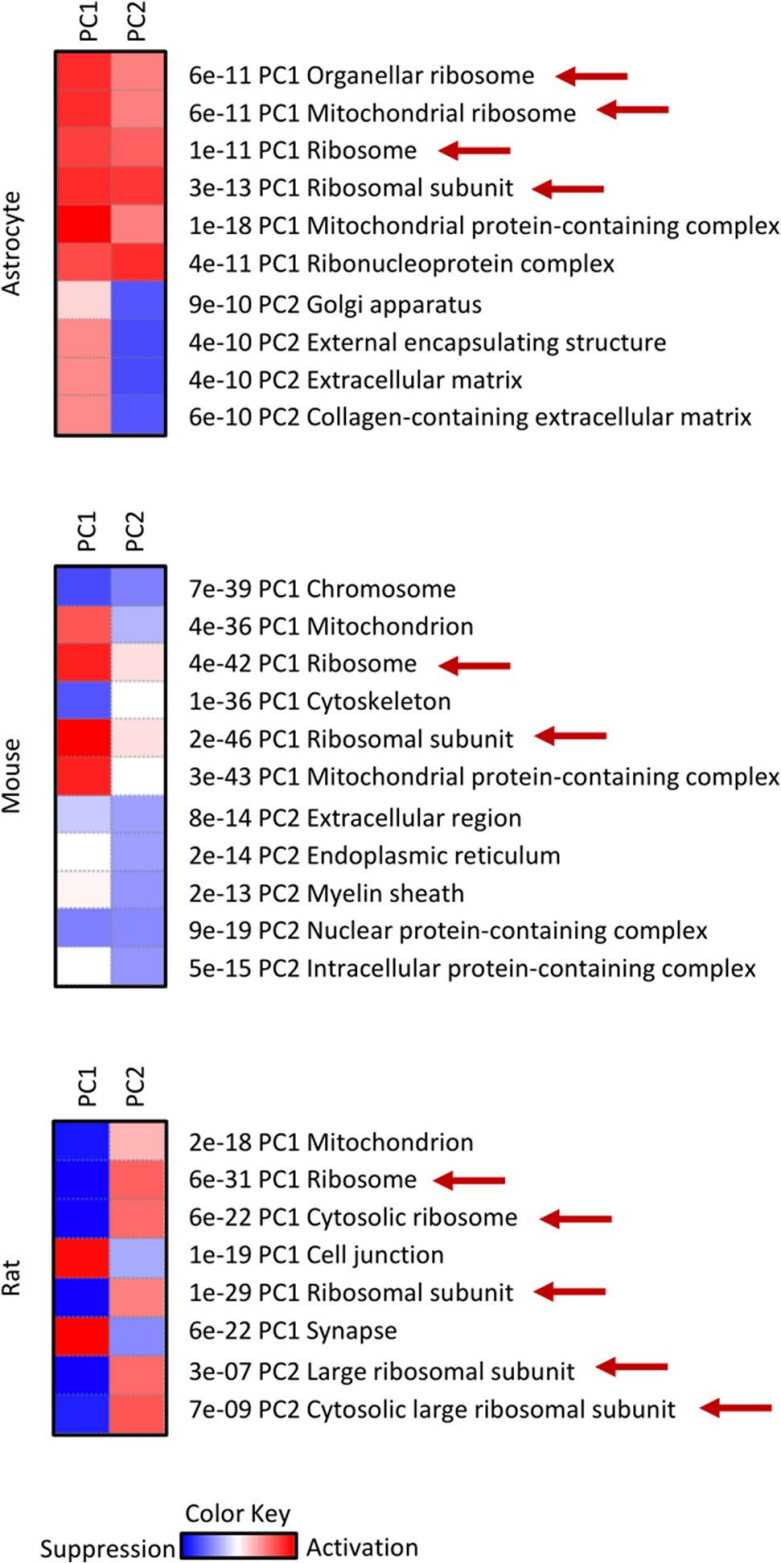
**PGSEA analysis considering GO cellular component gene set.** Red arrows indicate the GO: cellular component related to ribosomes. Red and blue in the square boxes indicate an activated pathway (corresponding to a positive *z*-score) and a suppressed pathway (corresponding to a negative z-score), respectively

The pre-process analyses and the parameters employed for all the datasets are reported in Fig. S1, S2, S3 and S4 and Table S1. The selection parameters filtered out 30–50% of the genes as mentioned in Table S2. The analysis involving the motor-like neuron model revealed a lack of correlation (Fig. S1, panels A-B) and also heterogenous distribution of the transformed data (Fig. S1, panel C) between samples of the same groups. Furthermore, the heatmap and hierarchical clustering tree built using the genes with maximum expression level at the top 75% did not achieve perfect discrimination among the groups (Fig. S1, panels D-E). Hence, the motor-like neuron model was excluded from the analyses. All other datasets showed high correlation and homogenous distribution of the transformed data among the various samples, with the reference groups and VitB12 deficiency mimicking groups distinctly grouped in the hierarchical clustering trees, although a little variation was observed among the technical replicates (Fig. S2, S3 and S4, panels A-E). Furthermore, the PCA for each dataset gave Principal Component (PC)1 and PC2 accounting for 55% and 28%, 63% and 16%, and 56% and 17% for the astrocyte, mouse and rat models, respectively, which led to cluster discrimination of the samples (Fig. S2, S3 and S4, panel F), whereas samples from the motor-like neuron model overlapped (Fig. 1S, panel F).

DEG analysis was performed on each dataset by comparing the VitB12 deficiency mimicking groups vs reference groups (see Table S1 for parameters and Fig. S5). The number of genes that were upregulated and downregulated were 191 and 308 in the astrocyte model, 844 and 132 in the mouse model and 880 and 1494 in the rat model, respectively (Fig. S5, panels A-C, left side). For each dataset, the gene expression levels of *Mmachc* and *Cd320* (encoding for metabolism of cobalamin associated C and transcobalamin receptor proteins, respectively), were reported, as they were the key factors involved in the metabolism of VitB12 (Table 2).

**Table 2:** Gene fold change from DEG, transciption factor gene targets (according to ChEA3) and peak score from THAP11 ChIP-Seq, in accord with Chern et al. (2022)

Pathway	Gene	Astrocyte		Mouse		Rat		Mouse TF gene targets			THAP11 (Peak score)
		log2 FC	adj.Pval	log2 FC	adj.Pval	log2 FC	adj.Pval	MYC	E2F1	Erg3	
VitB12 metabolism	*Mmachc*	− 0.14	0.67	− 2.35	4.75E − 62	− 0.28	0.21				633.6
	*Cd320*	− 0.32	0.40	− 0.16	0.27	0.94	0.021				–
Ribosome	*Rps15a*	− 0.30	0.40	− 0.66	1.43E − 10	0.37	0.06	+	+		23.2
	*Rps25*	− 0.20	0.48	− 0.77	5.76E − 13	0.36	0.07	+			–
	*Rps27a*	− 0.12	0.65	− 0.74	7.67E − 13	0.26	0.24	+	+		490.5
	*Mrpl14*	− 0.16	0.58	− 0.70	1.13E − 07	1.08	6.74E − 3			+	23.*5*
	*Rps24*	− 0.13	0.64	− 0.67	2.32E − 11	0.27	0.19	+	+	+	8.29
	*Mrpl20*	− 0.07	0.82	− 0.65	1.41E − 10	0.68	7.50E − 3				50.6
	*Rbm3*	0.07	0.84	− 0.60	1.46E − 07	− 0.05	0.82	+	+		48.6
	*Mrpl54*	− 0.06	0.84	− 0.60	9.79E − 06	0.65	0.02		+		306.9
	*Mrpl41*	− 0.06	0.87	− 0.81	2.24E − 15	0.14	0.47		+		–
	*Rpl12*	− 0.47	0.49	− 0.62	6.16E − 10	0.14	0.47	+	+		21.2
	*Rpl22l1*	− 0.06	0.92	− 0.90	1.65E − 13	0.06	0.74		+		4.57
	*Rpl35*	− 0.08	0.85	− 0.14	0.26	0.18	0.29	+	+		23.2
	*Chchd1*	− 0.41	0.25	− 0.35	8.05E − 03	− 0.25	0.16		+	+	–
	*Rpl13a*	− 0.10	0.72	− 1.31	8.73E − 04	0.49	0.03		+	+	−
	*Rpl41*	0.05	0.88	− 0.81	2.24E − 15	0.46	0.05		+	+	85.7
Transcription factor	*Myc*	− 0.34	0.37	0.12	0.39	0.40	0.34				5.36
	*E2f1/Necab3*	− 0.33	0.36	0.38	0.03	0.62	8.46E − 3				−
	*Egr3*	− 0.30	0.55	0.16	0.80	0.45	0.05				−
	*Thap11*	0.02	0.95	0.45	1.04E − 04	0.22	0.16				933.7
	*Hcfc1*	− 0.25	0.43	0.25	0.03	− 0.44	0.10				21.6

Results from DEG analysis were used to conduct the enrichment analysis considering the GO cellular component gene set (Fig. S5 and Table S3). A hierarchical clustering tree map describes the difference in distances among the similar-termed GO, exploiting the percentage of overlapped genes involved in each pathway (Fig. S5, panels A-C, right panel). The comparison among the 3 tree maps revealed that the ribosomal biology was affected only in the mouse and rat models (ribosomal pathways modified in both the animal models showed low values of adjusted P-values). Surprisingly, in the rat model, although the modulation of the ribosome pathway was evident, the analysis revealed an opposite trend (upregulation of gene expression) compared to that of the mouse model (the ribosomal genes being globally downregulated) (Fig. S5, panels A-C, right panel).

With the aim to corroborate these observations, a different analysis was performed on the datasets. The PGSEA, considering GO cellular component (PAGE algorithm) as a gene set, performed one-sample *t*-test on each gene specifically involved in a specific biological process branch of GO, thereby obtaining adjusted *P*-values that were used to rank the pathways contributing to the determination of each principal component (see PCA analysis, Fig. S2, S3 and S4, Panel F). This analysis was more sensitive and brought to light the weaker modifications. Therefore, Fig. 1 shows the observations from the PGSEA analysis for each dataset considered. For each pathway, the load of PC1 and PC2 on the pathway was indicated by the appropriate colours (red and blue indicate activated and suppressed pathways, respectively). The Pathways were also labelled with the false discovery rate (FDR). Both for the mouse and rat models, alteration of genes involved in the ribosome gene dataset participates in the load of the first component by being negatively or positively correlated, respectively. This result was in accord with the DEG analysis. Although the genes involved in the “Ribosome” GO negatively and weakly loaded the PC2 for the rat model, the PC1 accounted for 56% of the PCs, thereby portraying a prominent role. Surprisingly, the PGSEA analysis performed on the astrocyte dataset also revealed some ribosomal alterations within this model (Fig. 1).

A different analysis from DEG, called “Pathways,” was also performed. While DEG analysis is sensitive to arbitrary cut-offs, Pathways analysis helps in the identification of coherently altered pathways using fold-change values. On choosing the GAGE method and GO cellular component gene set, this analysis gave results very similar to that of the PGSEA analysis, thereby corroborating observations regarding the alterations in the ribosome pathways in all three datasets (Table S4).

### THAP11 targets analysis in mouse model

**Fig. 2:**
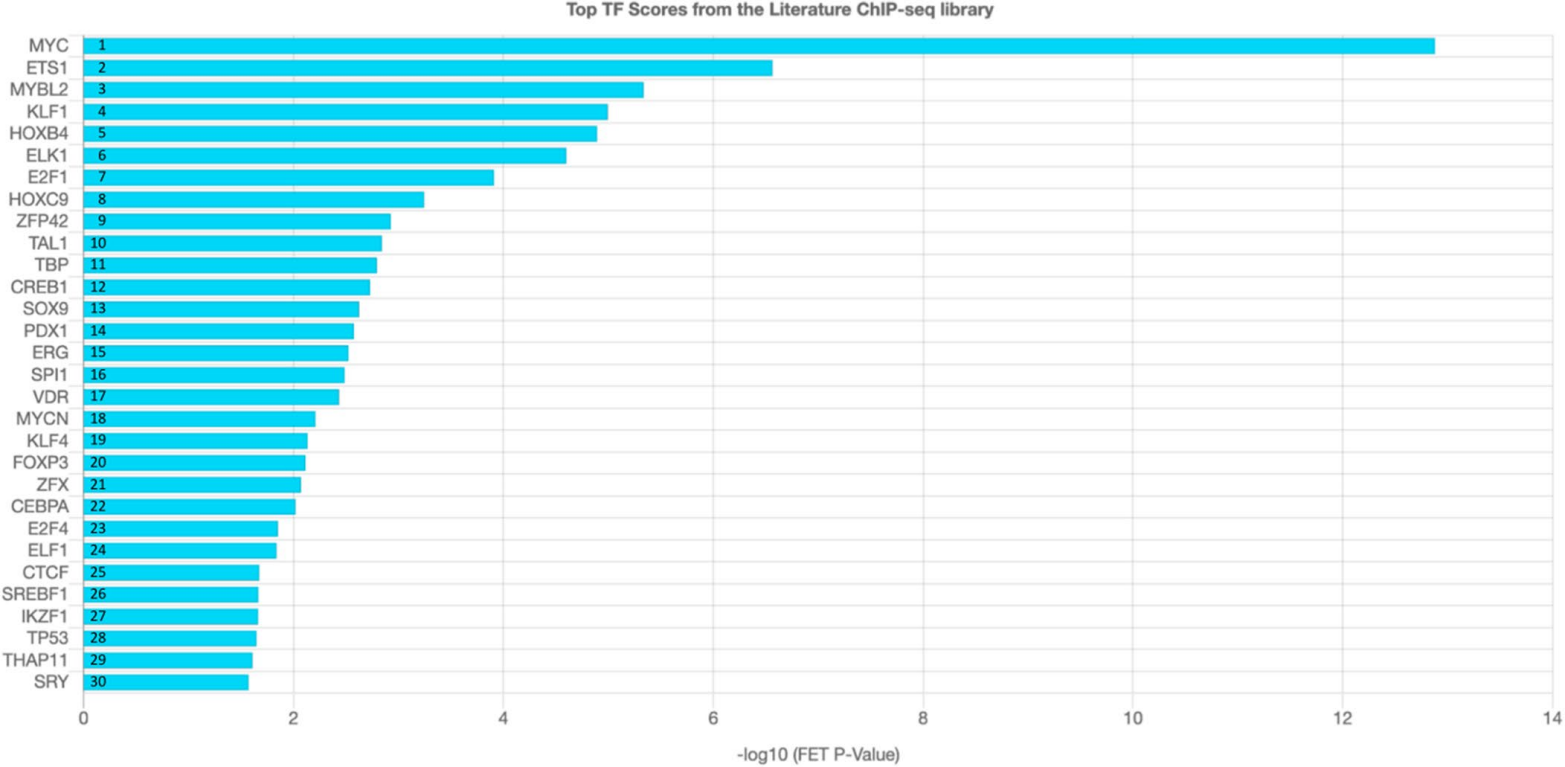
**ChIP-X enrichment analysis 3 (ChEA3).** The analysis ranks the TFs that modulate the genes considered to build the “Ribosome” pathway in the Enriched pathways DEG analysis on mice, considering the GO cellular component gene set. The name at the base of histograms indicates the TFs while the number in the histograms is their place in the rank. FET refers to Fisher’s exact test

Since the mouse model was obtained through genetic engineering and the other models by a nutritional deficiency of VitB12, it was questioned whether the alteration in “Ribosome” pathway was directly linked to THAP11 mutation. A ChEA3 analysis was performed on the genes that were considered to build the “Ribosome” pathway in the Enrichment analysis on mice from DEG results (obtained by considering the GO Cellular Component gene set) (Table S5). ChEA3 is a transcription factor enrichment analysis that ranks TFs associated with a specific list of genes. As a gene set library, “Literature” was chosen for the analysis. This library is built from the data of published experiments obtained from humans, mice and rats (Keenan et al. 2019). The analysis highlighted that THAP11 was ranked 29th place with an overlap of 4 genes, while MYC was the top-ranked one with 21 genes overlapping out of 34 (Table S6 and Fig. 2). Considering the genes that were included in “Ribosome” pathway common to both mice and rats (15 genes as reported in Table 2), only 2 of them were returned in the ChEA3 results. Furthermore, Chern and co-workers performed a ChIP sequencing analysis to establish the THAP11 target genes (Chern et al. 2022) and the peak scores (–log10 (*P*-value)) obtained were reported in Table 2. With the exception of *Rps27a* and *Mrpl54*, the other ribosomal genes had a low score. These results are in accord with ChEA3 analysis and collectively suggested that THAP11 did not have a global action on the ribosomal genes, but were limited to very specific ones.

Next, it was to be understood whether the alterations in astrocytes and rats were possibly linked with a change in the THAP11 expression that, in turn, can affect the genes involved in the metabolism of VitB12. Considering the ChIP-seq results regarding THAP11, a high peak score was observed for both *Mmachc* and, interestingly, for *Thap11*, thereby suggesting a positive feedback regulation mechanism for this transcription factor in the mouse model. However, *Thap11* and *Hcfc1* (which encodes for a transcription factor and together with THAP11 jointly regulates the *Mmachc* expression) gene levels were not altered in the astrocyte and rat models on inducing the VitB12 deficiency, thereby excluding any alteration linked to these genes.

### TF enrichment analysis in DEG

**Fig. 3:**
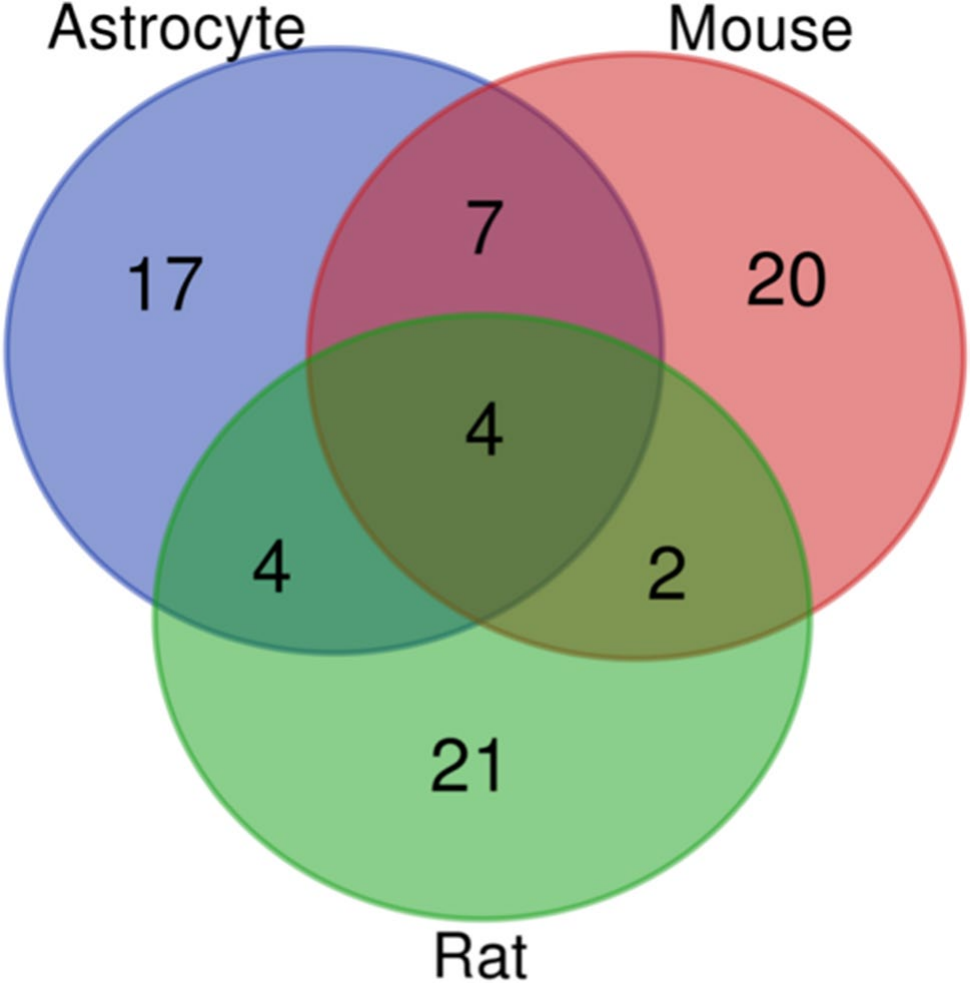
**Venn diagram on TF enrichment analysis.** The Venn diagram reports TF enrichment analysis, according to DEG analysis results, performed on all the models. The Venn diagram was made using https://bioinformatics.psb.ugent.be/webtools/Venn/

The observed difference in the ribosomal gene regulation for the models could be due to the transcription factors being regulated differently after the induction of VitB12 deficiency. Aiming at understanding this point, a TF Enrichment analysis in DEG was performed. This returns the more representative TFs that target the promoters of the genes involved in the phenom based on the greater variable ones without focusing on a specific pathway. Thus, although DEG analysis was unable to detect the alterations in ribosomal gene expression in the astrocyte model, the latter was included in the analysis. TF enrichment highlighted a complex panorama (Table S7). A possible overlap of TFs (without considering the direction, i.e., upregulation or downregulation) was evaluated in the results obtained from each group: as reported in the Venn diagram (Fig. 3) and Table 3, and four common TFs encoded by *Patz1/Zfp278*, *Sp4*, *Egr1* and *Zbtb7b* genes were found. Surprisingly, none of these TFs were returned in the ChEA3 analysis performed on ribosomal genes. Furthermore, the intersection of the TF Enrichment analysis performed for each dataset showed an enrichment of genes having E2F1 as TFBSs in the promoter common for the astrocyte and rat models and EGR3 common for the mouse and rat models. Interestingly, ChEA3 analysis highly ranked E2F1 and ERG3 when genes from “Ribosome” pathway from mice were used as a query, with an overlap of 18 genes and 9 genes respectively (Table S6).

**Table 3:** TF enrichment analysis on DEG results (Venn diagram intersection)

Set	No. of genes	Genes
Astrocyte ∩ Mouse ∩ Rat	4	*Patz1/Zfp278, Sp4, Egr1, Zbtb7b*
Astrocyte ∩ Mouse	7	*Zic5, Zfp281, Spic, Sfpi1, Zfp202, Zfp740, Plag1*
Astrocyte ∩ Rat	4	*Zic4, E2f1, Klf5, Plagl1*
Mouse ∩ Rat	2	*Sp1, Egr3*
Astrocyte	17	*Bhlha15, Zic2, Zic1, Tcf3, Elf1, Erg, Msc, Runx1, Mecp2, Tbx1, Foxh1, Ascl2, Myf6, E2f6, Zic3, Spib, Ets1*
Mouse	20	*Elf4, Etv1, Etv4, Sp100, Elk1, Elf5, IRC900814, Etv6, Etv3, Cenpb, Elf3, Elk4, Elf2, Etv5, Gm4881, Kdm2b, Gm5454, Pax4, Glis2, Elk3*
Rat	21	*D3ZXG8_RAT, Egr2, E2f3, Zfp161, Klf7, Klf14, Tcfap2d, LOC100363771, Gmeb2, Gmeb1, Sp6, Cxxc1, D3ZGU0_RAT, RGD1305899, Klf12, D3ZQL2_RAT, Klf4, D3ZLI0_RAT, Zfx, Egr4, Sp2*

### Transcription factors and “Ribosome” pathway gene expression

Considering the common TFs returned by the TF enrichment from the DEG and ChEA3 analyses, the gene levels of *E2f1* and *Erg3* for all the models were evaluated. Since MYC was highly ranked in the ChEA3 analysis for “Ribosome” pathway, *Myc* was also included (Table 2). A significant increase in the gene expression of *E2f1* under VitB12 deficiency mimicking conditions in the mouse and rat models (with the latter showing a greater induction) was observed, but not in the astrocyte model; however, the gene expression levels of *Erg3* and *Myc* were not altered. Furthermore, the expression of the genes common to the mouse and rat models that take part in the “Ribosome” pathway establishment were selected (Table S5) and for each of them, it was annotated if they were returned in the ChEA3 analysis (Table S6) and the log2 fold change (log2FC) (Table 2). Comparing the genes involved in the “Ribosome” pathway with adjusted *P*-values < 0.05, it was observed that all of them were downregulated for the mouse model (*Rps15a, Rps25, Rps27a, Mrpl14, Rps24, Rbm3, Mrpl54, Mrpl41, Rpl12, Rpl22l1, Chchd1, Rpl13a and Rpl41*), whereas the significantly modulated genes were upregulated for the rat model (*Mrpl14, Mrpl54, Rpl13a*). A majority of the above-mentioned genes were observed to be associated with E2F1 in the ChEA3 analysis (mainly based on data from mice). In accord with the mouse model, astrocytes showed a downregulation trend in ribosome-related genes, although it was observed to be insignificant.

### Putative transcription factor binding sites of the genes involved in the “Ribosome” pathway for rat model

Since E2F1 regulates the expression of ribosomal genes positively (Ayrault et al 2006; Gnanasundram and Fåhraeus 2018; van Riggelen et al 2010), it was evaluated whether E2F1 could occupy the promoter and modulate the upregulation of *Mrpl14*, *Mrpl54*, and *Rpl13a* genes observed in the rat model. The ChEA3 analysis performed on the genes involved in “Ribosome” pathway returned only one dataset for the rat model; therefore, an *in silico* analysis was needed to evaluate the putative TFBSs in rats.

For the promoter analysis, a region of 600 bps upstream of the gene was considered. Firstly, an alignment of both mRNA transcripts and promoter regions for each gene was performed for assessing the similarities in transcripts and gene promoter regions using Pairwise Sequence Alignment. A comparison of the mRNA transcripts of *Mrpl14*, *Mrpl54*, and *Rpl13a* from mice and rats gave a similarity and gaps of 67.3% and 29.1%, 81.0% and 8.6%, and 57.4% and 39.8%, respectively, thereby indicating a certain degree of structural relationship (Fig. S6, S7 and S8 and Table 4). The same analysis performed on the *Mrpl14*, *Mrpl54*, and *Rpl13a* promoters highlighted a similarity and gaps of 42.1% and 31.2%, 45.7% and 21.1%, and 48.1% and 25.8%, respectively, thereby suggesting a low structural conservation of the promoter regions due to the similarities being lower than 50% (Fig. S9, S10 and S11 and Table 4).

**Table 4:** Pairwise sequence alignment

Gene	mRNA		Promoter	
	Similarity %	Gaps %	Similarity %	Gaps %
*Rpl13a*	57.4	39.8	48.1	25.8
*Mrpl54*	81.0	8.6	45.7	21.1
*Mrpl14*	67.3	29.1	42.1	31.2
*Chchd1*	82.7	9.7	39.9	20.3
*Rbm3*	33.3	62.6	39.3	47.1

**Fig. 4:**
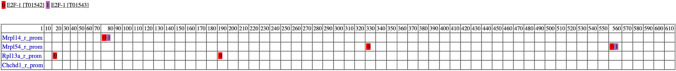
**PROMO analysis of promoters.** Factors predicted within a dissimilarity margin less or equal to 15%. The progressive numbers on the top of the grid indicate the distance from the 5′ of the DNA sequences considered

To investigate whether putative E2F1 TFBSs were present in the promoters of *Mrpl14*, *Mrpl54*, and *Rpl1* for the rat model, an *in silico* analysis was performed using PROMO, an online software which could predict TFBSs (Farré et al. 2003; Messeguer et al. 2002). This software highlighted multiple putative E2F1 TFBSs in the promoters of *Mrpl14*, *Mrpl54*, and *Rpl13a* for the rat model (Fig. 4 and Table 5). In spite of all the genes involved in “Ribosome” pathway exhibiting an upregulation trend for the rat model, the expression of *Chchd1* and *Rbm3* genes were reduced during the VitB12 deficiency mimicking conditions (Table 2). A comparison of the *Chchd1* and *Rbm3* mRNA transcripts for the mouse and rat models highlighted a similarity and gaps of 82.7% and 9.7% and 33.3% and 62.6%, respectively (Fig. S12 and S13, Table 4), whereas, with regard to the promoter regions, the similarity and gaps were observed to be 39.9% and 20.3% and 39.3% and 47.1%, respectively (Fig. S14 and S15 and Table 4). The pairwise sequence alignment analysis on *Rbm3* revealed very low similarity for the gene transcripts and also wide gaps for both the gene transcripts and promoter sequences, thereby making their comparison unreliable. Therefore, *Rbm3* was excluded from the subsequent analysis. Furthermore, PROMO analysis performed on the *Chchd1* promoter does not identify any putative E2F1 TFBSs, thereby hypothesizing that the observed downregulation could be associated with the absence of E2F1 TFBSs in the promoter of *Chchd1 gene*.

**Table 5:** PROMO analysis

Sequence name	Factor name	Start position	End position	Dissimilarity %	String
Mrpl14	E2F-1 [T01542]	71	77	5.13	CCCCGCT
	E2F-1 [T01543]	72	80	7.86	CCCGCTTCT
Mrpl54	E2F-1 [T01542]	320	326	8.03	CACCGCT
	E2F-1 [T01542]	553	559	7.88	GGCCGCG
	E2F-1 [T01542]	556	562	3.40	CGCGGGG
	E2F-1 [T01543]	553	561	7.22	GGCCGCGGG
Rpl13a	E2F-1 [T01542]	10	16	8.85	CGCGGTC
	E2F-1 [T01542]	188	194	9.61	GTCCGCT

## Discussion

The crucial role of VitB12 in brain homeostasis, including neurodevelopment and neurodegenerative/neurological diseases, is known, although the underlying mechanisms remain poorly understood. Therefore, with the aim of gathering new insights on the errors in VitB12 metabolism with specific reference to gene expression response, RNA-Seq and microarray datasets generated from experimental models involving VitB12 metabolism defects were considered in this study. Datasets were obtained from the GEO DataSets archive and by limiting the inquiry to CNS or CNS-related models, although a great heterogeneity in methods were employed to generate VitB12 mimicking deficiency conditions in cell and animal models, it was possible to identify four suitable datasets. Three of these datasets were analysed as part of previous publications (GSE99113, GSE161763, and GSE104164) (Chern et al. 2022; Jonnalagadda et al. 2022; Willekens et al. 2019), whereas any analysis regarding the last dataset (GSE103417) was not published, although the authors of the dataset submitted a manuscript reporting the same experimental design (Battaglia-Hsu et al. 2009).

The DEG analysis performed on each dataset compared the VitB12 deficiency mimicking groups vs reference groups. For each dataset, about 18,000 genes were taken into account. DEG analysis revealed a difference in the number of genes that were modulated: considering the upregulated or downregulated gene, astrocytes gave the lowest number (191 and 308, respectively), whereas, the rat model gave the highest (880 and 1494). Enrichment Pathways analysis, followed by the DEG analysis, highlighted alterations in the regulation of the genes involved in ribosomes as a consequence of the VitB12 metabolism modification, in both the animal models with opposite trends. Although different similar-termed GO pathways involving the ribosomes emerged (as underscored by hierarchical clustering tree maps), the “Ribosome” pathway from DEG analysis was used as a reference for subsequent investigations in this study. In the mouse model, a general downregulation of the ribosomal gene expression was observed, whereas, on the contrary, there was an upregulation of the ribosomal gene expression in the rat model. However, there were no significant alterations observed for mentioned pathways in the astrocyte model. With regard to the mouse astrocytes, it is possible that being a cellular model, the maintenance of the culture, e.g., periodically changing the culture medium, reduces the accumulation of toxic byproducts (such as homocysteine) and can mitigate the alterations due to VitB12 deficiency, thereby leading to a weak gene modulation (in accord with the lower number of altered genes detected) and hindering the identification of many emerging alterations in the ribosomal pathways from DEG analysis. Alternatively, since DEG analysis is based on variance, the experimental design of the astrocytes dataset (made up of 2 replicas for each condition) could affect results by flatting the possible differences between groups. As a result of more sensitive analyses of all models, including PGSEA and Pathways, a decrease in the expression of the ribosomal genes in astrocytes of a lesser magnitude was observed, thus confirming the hypothesis that culture maintenance attenuated the effects of VitB12 deficiency on the cellular model.

The defects arising primarily due to VitB12 deficiency or alteration in proteins linked to the VitB12 metabolism pathway could be exacerbated by a general downregulation of *Mmachc* or *Cd320*. Indeed, the downregulation or a defect in MMACHC, the pivotal enzyme in the metabolism of VitB12, and in the transcobalamin receptor (encoded by *Cd320*) are associated with VitB12-related defects (Chern et al. 2022; Fidaleo et al. 2021; Pappas et al 2022). Therefore, to characterize the models chosen for this study, *Mmachc* and *Cd320* gene expression levels were evaluated. A highly significant downregulation was observed for the mouse model, whereas the mouse astrocyte and rat models showed a small downregulation of *Mmachc*, however not significant. With regard to *Cd320*, a reduction in the gene expression level was observed in the mouse and astrocyte models (although without significance), but not in the rat model. Such observations could be plausible, considering the approaches used to induce VitB12 deficiency: in the mouse model, *Mmachc* expression was altered following mutation in the THAP11 transcription factor which participates in the *Mmachc* gene regulation; whereas, VitB12 deficiency in the astrocyte and rat models was induced by removing VitB12 from the food or culture medium which does not lead to an alteration in the expression of *Mmachc*, at least under the tested conditions (Battaglia-Hsu et al. 2009; Chern et al. 2022; Jonnalagadda et al. 2022; Willekens et al. 2019).

The selected models included in the present study were very heterogenic considering the cell types (astrocytes, neuroblastoma, brain and cerebellum) and the species (mouse and rat) (Table 1). Furthermore, in the mouse astrocyte and rat models, the deficiency was induced by removing VitB12 from the culture medium or food, whereas the mouse model was obtained by genetic manipulations of *Thap11,* thus resulting in a downregulation of *Mmachc*. THAP11^F80L/F80L^ mice exhibited a complex disease phenotype including craniofacial deformity. Despite the controversy, defects in cranial formation and VitB12 metabolism may be linked. Chern and colleagues generated a mouse model overexpressing *Mmachc* — in the presence of THAP11^F80L/F80L^ — in the neural crest cells (which differentiated into a portion of the craniofacial skeleton) showing normal cranial formation thus demonstrating that the impairment in bone development was not due to VitB12 metabolism defect, but mainly related to the other THAP11 target genes. On the contrary, a direct involvement of cobalamin metabolism in cranial development was evident in the zebrafish model (Quintana et al. 2014). Currently, an exhaustive description of the involvement of VitB12 defects and cranial formation is unavailable (Chern et al. 2022; Quintana et al. 2014). Furthermore, regarding the THAP11^F80L/F80L^ mouse model, Chern and colleagues observed ribosomopathy and postulated that it was mainly linked to THAP11 rather than MMACHC alteration. Even though they concluded that the mechanism of action of THAP11 (and HCFC1) in ribosome biogenesis remains unsolved (for instance they observed tissue-specific phenotypes), their observations from the ChIP sequencing suggested a direct binding of THAP11 to some of the ribosomal gene promoters (Chern et al. 2022). Since an overall modification was observed in the expression of genes linked to ribosomes in all the datasets chosen for this study, the possible occupancy of THAP11 in the promoter of “Ribosome” pathway genes, that were commonly modulated in all the models, was evaluated by assessing the peak scores from the ChIP sequencing results from Chern and colleagues (Chern et al. 2022). A high peak score was observed for only two genes involved in ribosome biogenesis in mice. Therefore, considering the ChEA3 analysis – many other TFs are higher ranked compared to THAP11, including the MYC family which are considered as the master regulators of ribosome biogenesis (Arabi et al. 2005; Boon et al. 2001; Cole and Cowling 2008; Grandori et al. 2005; Grewal et al 2005; van Riggelen et al. 2010) — and considering the common overall misregulation of the ribosomal genes in all the datasets considered in this study, it is possible to hypothesize that the modulation of genes related to the “Ribosome” pathway was not strictly associated to the THAP11 mutation, but more generally due to the VitB12 metabolism alteration.

In order to identify a common TF for all the models that could be responsible for the ribosomal gene regulation associated with the VitB12 metabolism alteration and more specifically, the one responsible for the divergence in the mouse and rat models, a TFs enrichment analysis was performed. The analysis took into account the genes that were globally upregulated and downregulated (without focusing on specific gene clusters) and highlighted four common TFs, for all the models, encoded by *Patz1/Zfp278*, *Sp4*, *Egr1* and *Zbtb7b* genes that were not returned by the ChEA3 analysis performed on ribosomal genes (the latter is mostly based on the datasets from mouse). This suggested that the above-mentioned TFs could take part in a more general gene regulation process and not be related to the “Ribosome” pathway alteration, at least for the mouse model. Interestingly, E2F1 and EGR3 emerged as TFs modulated following the VitB12-metabolic defect (not common to all models) and are returned by the ChEA3 analysis, thereby providing a hint about their involvement in the ribosomal gene regulation in all the models. Furthermore, the ChEA3 analysis revealed MYC as the highest-ranked TF which modulated the ribosome genes selected. As reported in Table 2, E2F1 was associated with the majority of the selected genes as compared to MYC and EGR3, at least for the mouse model. In mice, the fact that the Enrichment TF analysis did not allow to emerge E2F1 could be linked to several features associated with the model and the analysis: (i) Enrichment TF analysis is based on the more altered genes; (ii) astrocyte and rat models are obtained by inducing nutritional VitB12 deficiency; and (iii) in mice, THAP11 mutation can have a lower and indirect role in “Ribosome” pathway and a prominent direct role on several other different pathways. These can lead to the detection of ribosome gene alteration without highlighting the change in E2F1 TFBSs enrichment in mice. Thus, a different analysis approach was needed.

The regulation of a TF target gene can be regulated by its TF content. Hence, we evaluated the gene expression levels of *Myc, E2f1/Necab3* and *Egr3* and the expression levels of *E2f1/Necab3* were observed to be significantly upregulated in both the animal models, although rats showed a greater increase and higher significant upregulation due to the induction of the VitB12 metabolism defect. In spite of having very little information regarding ERG3 and its role in ribosomal gene regulation, the involvement of E2F1 and MYC in the positive regulation of ribosomal gene expression is quite known (Ayrault et al. 2006; Gnanasundram and Fåhraeus 2018; van Riggelen et al. 2010). The fact that E2F1 was greatly upregulated in rats as compared to that of mice after the induction of the VitB12-metabolic defect suggested its possible role in the divergent modulation of the ribosomal genes.

**Fig. 5:**
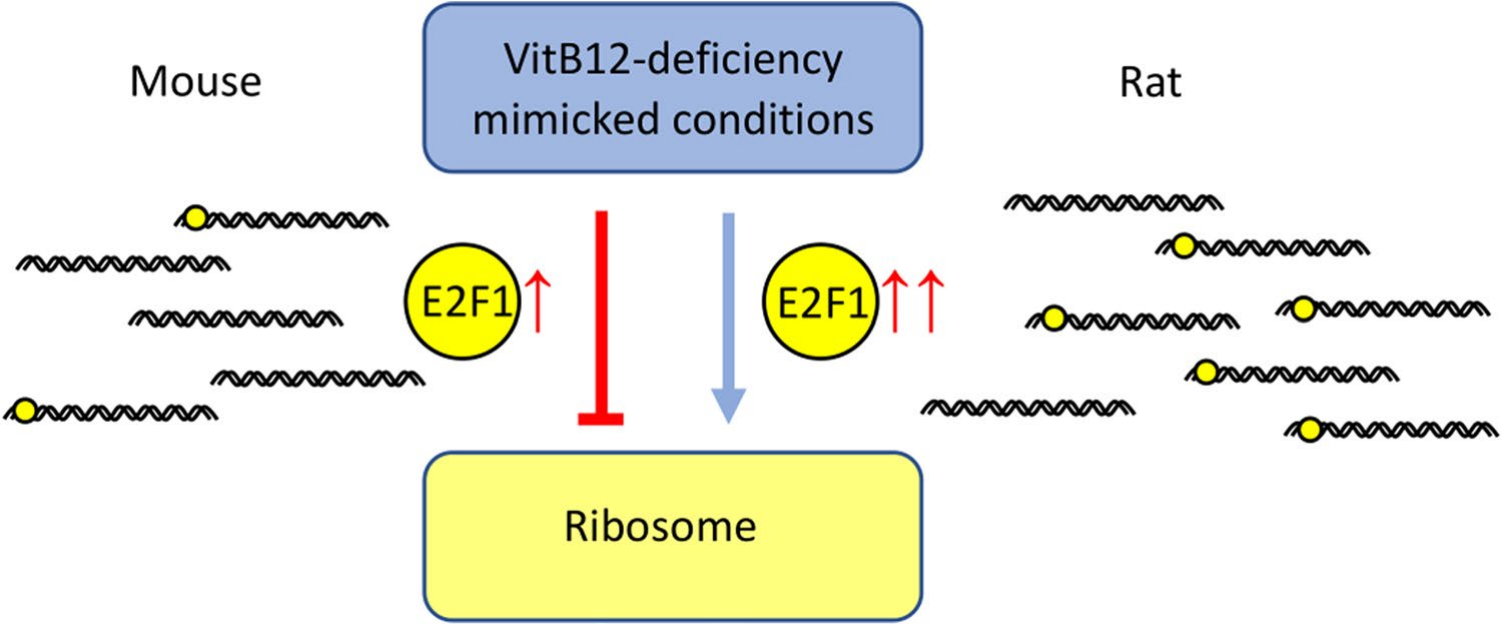
**Molecular hypothesis.** VitB12 deficiency leads to an upregulation of E2f1 in animal models (with rats showing a higher level). In rats, some genes involved in the ribosome pathway show putative E2F1 TFBSs. The greater expression of E2F1 and a higher TFBSs frequency in ribosome genes could sustain the upregulation of ribosome-related genes observed in rats

To reinforce the hypothesis on the possible involvement of *E2f1* in the divergent regulation of ribosomal genes in mice and rats, the promoter sequence of genes involved in the “Ribosome” pathway exhibiting an opposed behaviour were taken into account: these were *Mrpl14*, *Mrpl54* and *Rpl13a*. Before examining the promoter region, the transcripts of these genes were compared to establish a certain similarity, thereby allowing a genuine comparison. All the gene transcripts exhibited a similarity of over 50% and gaps below 50%, indicating an overall similar structure. The same analysis performed on the promoters revealed a lower similarity between mice and rats, thus underscoring a difference for the considered DNA portions. On comparing the promoter and transcript alignment results, it can be inferred that an evolutionary modification in the promoter could be responsible for the difference in gene regulation. Interestingly, using PROMO analysis, putative E2F1 TFBSs were predicted in the rat promoters. As a result of this and together with the higher *E2f1* gene expression, it can be concluded that rats had a greater amount of E2F1 protein which could bind *Mrpl14*, *Mrpl54* and *Rpl13a,* thereby activating their upregulation. To provide more evidence regarding this mechanism, the same analyses were performed on *Chchd1* and *Rbm3*, which were part of the “Ribosome” pathway and were both observed to be downregulated in rats after the induction of VitB12 deficiency. However, only *Chchd1* exhibited similarity after performing the alignment, thus allowing genuine comparison. PROMO analysis did not show any E2F1 TFBSs in the *Chchd1* promoter, thereby suggesting its non-involvement in E2F1 regulation. This could explain the *Chchd1* downregulation in rats after the induction of the VitB12-metabolic defect, irrespective of the increase in *E2f1* (Fig. 5).

The alteration of the ribosome-related gene expression can affect ribosome biogenesis leading to ribosomopathies. Although ribosomes are essential organelles, the manifestation of the defects is extremely cell- and tissue-specific and includes mRNA translation, cell cycle control and signalling pathways (e.g., TP53, MYC and mTOR) (Armistead and Triggs-Raine 2014). In general, non-dividing differentiated cells have a reduced ribosome biogenesis rate, whereas post-mitotic neurons demonstrate an intense rate of ribosome biogenesis, particularly in neurites, for the increased synthesis of local proteins to promote neuronal morphogenesis. Increased ribosome biogenesis can be achieved via the ERK1/2 signalling cascade by neurotrophic factors, including brain-derived neurotrophic factors (BDNF). Additionally, data suggest a strong relationship between the increase in ribosome biogenesis and neurite outgrowth during nerve regeneration following injury (Turi et al. 2019). Of note, ERK1/2 and E2F1 are involved in the same pathway regulating cell cycling (Korotayev et al. 2008), thus speculating a possible association of ribosome biogenesis in CNS to be validated.

In this study, the presented comparative analysis revealed ribosome-related gene modulation specific to cell and animal models and suggested the important role played by E2F1 in maintaining ribosome homeostasis in the CNS on inducing VitB12 deficiency mimicking conditions. Overall, literature data and the results reported from this study indicate ribosome biogenesis/E2F1 as a potential target for reversing the VitB12 deficiency side effects.

## Conclusion

There are ample pieces of evidence that suggest the role of VitB12 in CNS homeostasis, however, the precise mechanisms regarding the impairments due to its deficiency have not been elucidated. In this study, the available gene expression datasets with gene misregulation, following the induction of VitB12 metabolic defects, were considered to evaluate the possible underlying mechanisms and the mode of action. An alteration in the ribosome regulation was highlighted in all the models. The huge differences among the models and the protocols for mimicking the VitB12 deficiency considered in this study, strengthen the hypothesis of a conserved and basal mechanism involved in the regulation of ribosomal gene expression related to VitB12 metabolism. Furthermore, a possible role in the above-mentioned ribosomal regulation can be ascribed to the transcription factor E2F1 and cognate TFBSs. Although this study requires further validation employing the wet laboratory procedures, the results drawn from this comparative dataset analysis have provided new insights into the basic molecular mechanisms that might be involved in CNS homeostasis regarding its association with VitB12 deficiency.

## Supplementary Information

Below is the link to the electronic supplementary material.

Fig. S1 Inherited disorders of cobalamin metabolism disrupt nucleocytoplasmic transport of mRNA through impaired methylation/phosphorylation of HuR. (**A**) Scatter plot of the first two samples. (**B**) Correlation matrix reporting Pearson’s correlation coefficients. (**C**) Distribution of transformed data. (**D**) Heatmap of 1000 most variable genes. (**E**) Hierarchical clustering tree. (**F**) PCA analyses. d_WT: differentiated WT N1E-115 cells; d_TO: differentiated N1E-115 cells expressing TO chimeric proteins; d_OT: differentiated N1E-115 cells expressing OT chimeric proteins (PNG 540 kb)

High Resolution Image (TIFF 424 kb)

Fig. S2 RNA-seq for understanding the effects of vitamin B12 removal on astrocyte culture. (**A**) Scatter plot of the first two samples. (**B**) Correlation matrix reporting Pearson’s correlation coefficients. (**C**) Distribution of transformed data. (**D**) Heatmap of 1000 most variable genes. (**E**) Hierarchical clustering tree. (**F**) PCA analyses. B12_def: astrocytes cultured in free VitB12 medium; B12_suf: astrocytes cultured in normal medium (PNG 432 kb)

High Resolution Image (TIFF 346 kb)

Fig. S3 CblX disease is both an inborn error of cobalamin metabolism and a ribosomopathy. (**A**) Scatter plot of the first two samples. (**B**) Correlation matrix reporting Pearson’s correlation coefficients. (**C**) Distribution of transformed data. (**D**) Heatmap of 1000 most variable genes. (**E**) Hierarchical clustering tree. (**F**) PCA analyses. ctrl: wild-type mouse; mutant: mouse carrying THAP11^F80L/F80L^ mutated protein (PNG 418 kb)

High Resolution Image (TIFF 345 kb)

Fig. S4 Wnt-signaling pathways are dysregulated in female cerebellum following an early methyl donor deficiency in a rat nutritional model. (**A**) Scatter plot of the first two samples. (**B**) Correlation matrix reporting Pearson’s correlation coefficients. (**C**) Distribution of transformed data. (**D**) Heatmap of 1000 most variable genes. (**E**) Hierarchical clustering tree. (**F**) PCA analyses. CTRL: rats under standard diet; MDD_C: rats under diet deficient in folate (VitB9) and VitB12 and lowered in choline (PNG 543 kb)

High Resolution Image (TIFF 428 kb)

Fig. S5 Differentially Expressed Genes analysis (DEG). Heatmap (left panel) and hierarchical clustering tree (right panel) from DEG analysis considering GO Cellular Component gene set from astrocyte (**A**), mouse (**B**) and rat (**C**) models. Green dots in hierarchical clustering tree indicate a downregulated set of genes involved in a specific pathway, while red dots indicate upregulated ones. Numbers before the name of gene dataset is an adjusted p-value. The black square indicates the GO: Cellular component related to ribosomes (PNG 1036 kb)

High Resolution Image (TIFF 820 kb)

Fig. S6 Pairwise Sequence Alignment of *Mrpl14* mRNA from rat and mouse (PNG 446 kb)

High Resolution Image (TIFF 369 kb)

Fig. S7 Pairwise Sequence Alignment of *Mrpl54* mRNA from rat and mouse (PNG 367 kb)

High Resolution Image (TIFF 309 kb)

Fig. S8 Pairwise Sequence Alignment of *Rpl11a* mRNA from rat and mouse (PNG 539 kb)

High Resolution Image (TIFF 443 kb)

Fig. S9 Pairwise Sequence Alignment of *Mrpl14* promoter sequence from rat and mouse (PNG 387 kb)

High Resolution Image (TIFF 308 kb)

Fig. S10 Pairwise Sequence Alignment of *Mrpl54* promoter sequence from rat and mouse (PNG 379 kb)

High Resolution Image (TIFF 304 kb)

Fig. S11 Pairwise Sequence Alignment of *Rpl13a* promoter sequence from rat and mouse (PNG 379 kb)

High Resolution Image (TIFF 304 kb)

Fig. S12 Pairwise Sequence Alignment of *Chchd1* mRNA from rat and mouse (PNG 363 kb)

High Resolution Image (TIFF 308 kb)

Fig. S13 Pairwise Sequence Alignment of *Rbm3* mRNA from rat and mouse (PNG 927 kb)

High Resolution Image (TIFF 730 kb)

Fig. S14 Pairwise Sequence Alignment of *Chchd1* promoter sequence from rat and mouse (PNG 370 kb)

High Resolution Image (TIFF 297 kb)

Fig. S15 Pairwise Sequence Alignment of *Rbm3* promoter sequence from rat and mouse (PNG 390 kb)

High Resolution Image (TIFF 309 kb)

ESM 16 (XLSX 10 kb)

ESM 17 (XLSX 8 kb)

ESM 18 (XLSX 12 kb)

ESM 19 (XLSX 12 kb)

ESM 20 (XLSX 9 kb)

ESM 21 (XLSX 12 kb)

ESM 22 (XLSX 13 kb)

ESM 23 (XLSX 15 kb)

## Data Availability

The datasets analysed during the current study are available at the Gene Expression Omnibus (GEO) database on the NCBI website. GSE103417: https://www.ncbi.nlm.nih.gov/geo/query/acc.cgi?acc=GSE103417; GSE99113: https://www.ncbi.nlm.nih.gov/geo/query/acc.cgi?acc=GSE99113; GSE161763: https://www.ncbi.nlm.nih.gov/geo/query/acc.cgi?acc=GSE161763; GSE104164: https://www.ncbi.nlm.nih.gov/geo/query/acc.cgi?acc=GSE104164.

## References

[CR1] Allen LH (2012). Vitamin B-12. Adv Nutr.

[CR2] Andrès E, Vidal-Alaball J, Federici L, Loukili NH, Zimmer J, Kaltenbach G (2007). Clinical aspects of cobalamin deficiency in elderly patients. Epidemiology, causes, clinical manifestations, and treatment with special focus on oral cobalamin therapy. Eur J Int Med.

[CR3] Arabi A, Wu S, Ridderstråle K, Bierhoff H, Shiue C, Fatyol K, … Wright APH (2005). c-Myc associates with ribosomal DNA and activates RNA polymerase I transcription. Nat Cell Biol 7(3):303–310. 10.1038/ncb1225 10.1038/ncb1225 15723053

[CR4] Armistead J, Triggs-Raine B (2014). Diverse diseases from a ubiquitous process: the ribosomopathy paradox. FEBS Lett.

[CR5] Ata F, Bint I, Bilal A, Javed S, Shabir Chaudhry H, Sharma R, Fatima Malik R, … Bhaskaran Kartha A (2020). Optic neuropathy as a presenting feature of vitamin B-12 deficiency: a systematic review of literature and a case report. Ann Med Surg 60:316–322. 10.1016/j.amsu.2020.11.010 10.1016/j.amsu.2020.11.010 PMC7653199 33204422

[CR6] Ayrault O, Andrique L, Séité P (2006). Involvement of the transcriptional factor E2F1 in the regulation of the rRNA promoter. Exp Cell Res.

[CR7] Battaglia-Hsu S-F, Akchiche N, Noel N, Alberto JM, Jeannesson E, Orozco-Barrios CE, … Guéant JL (2009). Vitamin B12 deficiency reduces proliferation and promotes differentiation of neuroblastoma cells and up-regulates PP2A, proNGF, and TACE. Proc Natl Acad Sci USA 106(51):21930–21935. 10.1073/pnas.0811794106 10.1073/pnas.0811794106 PMC2788478 19959661

[CR8] Battaglia-Hsu S-F, Ghemrawi R, Coelho D, Dreumont N, Mosca P, Hergalant S, … Guéant J-L (2018). Inherited disorders of cobalamin metabolism disrupt nucleocytoplasmic transport of mRNA through impaired methylation/phosphorylation of ELAVL1/HuR. Nucl Acids Res 46(15):7844–7857. 10.1093/nar/gky634 10.1093/nar/gky634 PMC6125644 30016500

[CR9] Boon K, Caron HN, Van Asperen R, Valentijn L, Hermus MC, Van Sluis P, … Versteeg R (2001) N-myc enhances the expression of a large set of genes functioning in ribosome biogenesis and protein synthesis. EMBO J 20(6):1383–1393. 10.1093/emboj/20.6.1383 10.1093/emboj/20.6.1383 PMC145518 11250904

[CR10] Calderón-Ospina CA, Nava-Mesa MO (2020). B Vitamins in the nervous system: Current knowledge of the biochemical modes of action and synergies of thiamine, pyridoxine, and cobalamin. CNS Neurosci Ther.

[CR11] Chern T, Achilleos A, Tong X, Hill MC, Saltzman AB, Reineke LC … Poché RA (2022). Mutations in Hcfc1 and Ronin result in an inborn error of cobalamin metabolism and ribosomopathy. Nat Commun 13(1):134. 10.1038/s41467-021-27759-7 10.1038/s41467-021-27759-7 PMC8748873 35013307

[CR12] Cole MD, Cowling VH (2008). Transcription-independent functions of MYc: regulation of translation and DNA replication. Nat Rev Mol Cell Biol.

[CR13] Duncan ID, Radcliff AB, Heidari M, Kidd G, August BK, Wierenga LA (2018). The adult oligodendrocyte can participate in remyelination. Proc Natl Acad Sci USA.

[CR14] Farré D, Roset R, Huerta M, Adsuara JE, Roselló L, Albà MM, Messeguer X (2003). Identification of patterns in biological sequences at the ALGGEN server: PROMO and MALGEN. Nucl Acids Res.

[CR15] Fidaleo M, Tacconi S, Sbarigia C, Passeri D, Rossi M, Tata AM, Dini L (2021). Current nanocarrier strategies improve vitamin b12 pharmacokinetics, ameliorate patients’ lives, and reduce costs. Nanomaterials.

[CR16] Ge SX, Son EW, Yao R (2018). iDEP: An integrated web application for differential expression and pathway analysis of RNA-Seq data. BMC Bioinformatics.

[CR17] Gnanasundram SV, Fåhraeus R (2018). Translation stress regulates ribosome synthesis and cell proliferation. Int J Mol Sci.

[CR18] Grandori C, Gomez-Roman N, Felton-Edkins ZA, Ngouenet C, Galloway DA, Eisenman RN, White RJ (2005). c-Myc binds to human ribosomal DNA and stimulates transcription of rRNA genes by RNA polymerase I. Nat Cell Biol.

[CR19] Grewal SS, Li L, Orian A, Eisenman RN, Edgar BA (2005). Myc-dependent regulation of ribosomal RNA synthesis during Drosophila development. Nat Cell Biol.

[CR20] Henríquez P, Doreste J, Deulofeu R, Fiuza MD, Serra-Majem L (2007). Nutritional determinants of plasma total homocysteine distribution in the Canary Islands. Eur J Clin Nutr.

[CR21] Huemer M, Diodato D, Schwahn B, Schiff M, Bandeira A, Benoist J-F, … Garcia-Cazorla A (2017). Guidelines for diagnosis and management of the cobalamin-related remethylation disorders cblC, cblD, cblE, cblF, cblG, cblJ and MTHFR deficiency. J Inherit Metab Disease 40(1):21–48. 10.1007/s10545-016-9991-4 PMC5203859 27905001

[CR22] Jonnalagadda D, Kihara Y, Groves A, Ray M, & Saha A (2022). FTY720 requires vitamin B 12 -TCN2-CD320 signaling in astrocytes to reduce disease in an animal model of multiple sclerosis. BioRxiv. Retrieved from 10.1101/2022.01.10.475450v1.abstract 10.1016/j.celrep.2023.113545 PMC11066976 38064339

[CR23] Keenan AB, Torre D, Lachmann A, Leong AK, Wojciechowicz ML, Utti V, … Ma’ayan A (2019). ChEA3: transcription factor enrichment analysis by orthogonal omics integration. Nucl Acids Res 47(W1):W212–W224. 10.1093/nar/gkz446 10.1093/nar/gkz446 PMC6602523 31114921

[CR24] Korotayev K, Chaussepied M, Ginsberg D (2008). ERK activation is regulated by E2F1 and is essential for E2F1-induced S phase entry. Cell Signal.

[CR25] Kräutler B (2012). Biochemistry of B12-cofactors in human metabolism. Subcell Biochem.

[CR26] Messeguer X, Escudero R, Farré D, Núñez O, Martínez J, Albà MM (2002). PROMO: Detection of known transcription regulatory elements using species-tailored searches. Bioinformatics.

[CR27] Nielsen MJ, Rasmussen MR, Andersen CBF, Nexø E, Moestrup SK (2012). Vitamin B 12 transport from food to the body’s cells - a sophisticated, multistep pathway. Nat Rev Gastroenterol Hepatol.

[CR28] Nishimoto S, Tanaka H, Okamoto M, Okada K, Murase T, Yoshikawa H (2015). Methylcobalamin promotes the differentiation of Schwann cells and remyelination in lysophosphatidylcholine-induced demyelination of the rat sciatic nerve. Front Cell Neurosci.

[CR29] Pappas KB, Younan M, Conway R (2022). Transcobalamin receptor deficiency in seven asymptomatic patients ascertained through newborn screening. Am J Med Genet A.

[CR30] Quandt K, Frech K, Karas H, Wingender E, Werner T (1995). Matlnd and Matlnspector: new fast and versatile tools for detection of consensus matches in nucleotide sequence data. Nucl Acids Res.

[CR31] Quintana AM, Geiger EA, Achilly N, Rosenblatt DS, Maclean KN, Stabler SP, … Shaikh TH (2014). Hcfc1b, a zebrafish ortholog of HCFC1, regulates craniofacial development by modulating mmachc expression. Dev Biol 396(1):94–106. 10.1016/j.ydbio.2014.09.026 10.1016/j.ydbio.2014.09.026 PMC4391465 25281006

[CR32] Salinas M, Flores E, López-Garrigós M, Leiva-Salinas C (2018). Vitamin B12 deficiency and clinical laboratory: lessons revisited and clarified in seven questions. Int J Lab Hematol.

[CR33] Sangle P, Sandhu O, Aftab Z, Anthony AT, & Khan S (2020). Vitamin B12 supplementation: preventing onset and improving prognosis of depression. Cureus, 12(10). 10.7759/cureus.11169 10.7759/cureus.11169 PMC7688056 33251075

[CR34] Turi Z, Lacey M, Mistrik M, Moudry P (2019). Impaired ribosome biogenesis: mechanisms and relevance to cancer and aging. Aging.

[CR35] van Riggelen J, Yetil A, Felsher DW (2010). MYC as a regulator of ribosome biogenesis and protein synthesis. Nat Rev Cancer.

[CR36] Watanabe F, Bito T (2018). Vitamin B12 sources and microbial interaction. Exp Biol Med.

[CR37] Watkins D, Rosenblatt DS (2011). Inborn errors of cobalamin absorption and metabolism. Am J Med Genet C Semin Med Genet.

[CR38] Willekens J, Hergalant S, Pourié G, Marin F, Alberto JM, Georges L, … Dreumont N (2019) Wnt signaling pathways are dysregulated in rat female cerebellum following early methyl donor deficiency. Mol Neurobiol 56(2):892–906. 10.1007/s12035-018-1128-3 10.1007/s12035-018-1128-3 29804229

