# Supplementary figures and images for: Altered vitamin B12 metabolism in the central nervous system is associated with the modification of ribosomal gene expression: new insights from comparative RNA dataset analysis
Source: Funct Integr Genomics. 2023 Jan 23;23(1):45. doi: 10.1007/s10142-023-00969-6 (PMC9868042; doi:10.1007/s10142-023-00969-6)

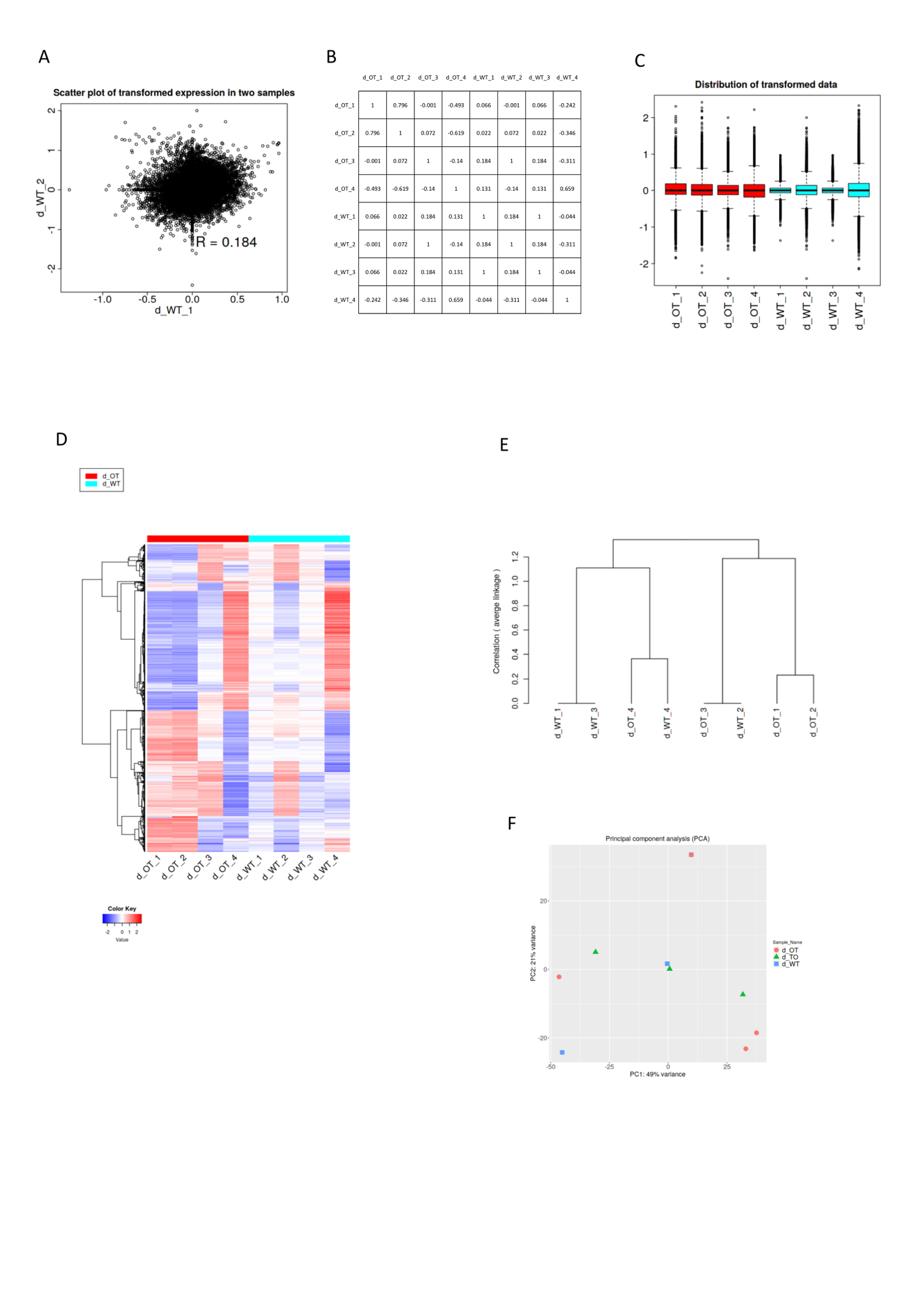

Supplement: Supplementary file 1 — Inherited disorders of cobalamin metabolism disrupt nucleocytoplasmic transport of mRNA through impaired methylation/phosphorylation of HuR. (A) Scatter plot of the first two samples. (B) Correlation matrix reporting Pearson’s correlation coefficients. (C) Distribution of transformed data. (D) Heatmap of 1000 most variable genes. (E) Hierarchical clustering tree. (F) PCA analyses. d_WT: differentiated WT N1E-115 cells; d_TO: differentiated N1E-115 cells expressing TO chimeric proteins; d_OT: differentiated N1E-115 cells expressing OT chimeric proteins (PNG 540 kb) [file 10142_2023_969_Fig6_ESM.png]

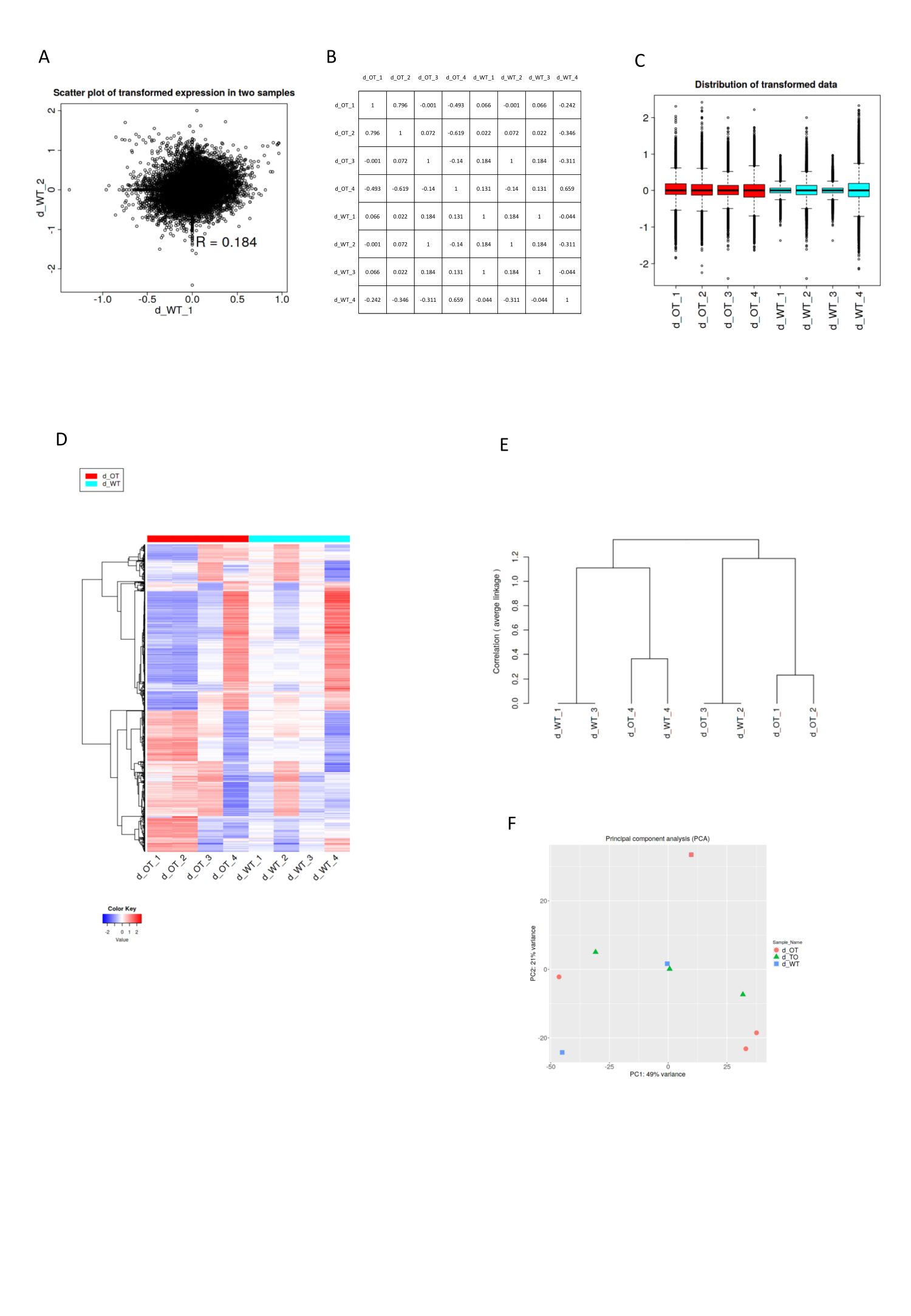

Supplement: Supplementary file 2 — High Resolution Image (TIFF 424 kb) [file 10142_2023_969_MOESM1_ESM.tiff]

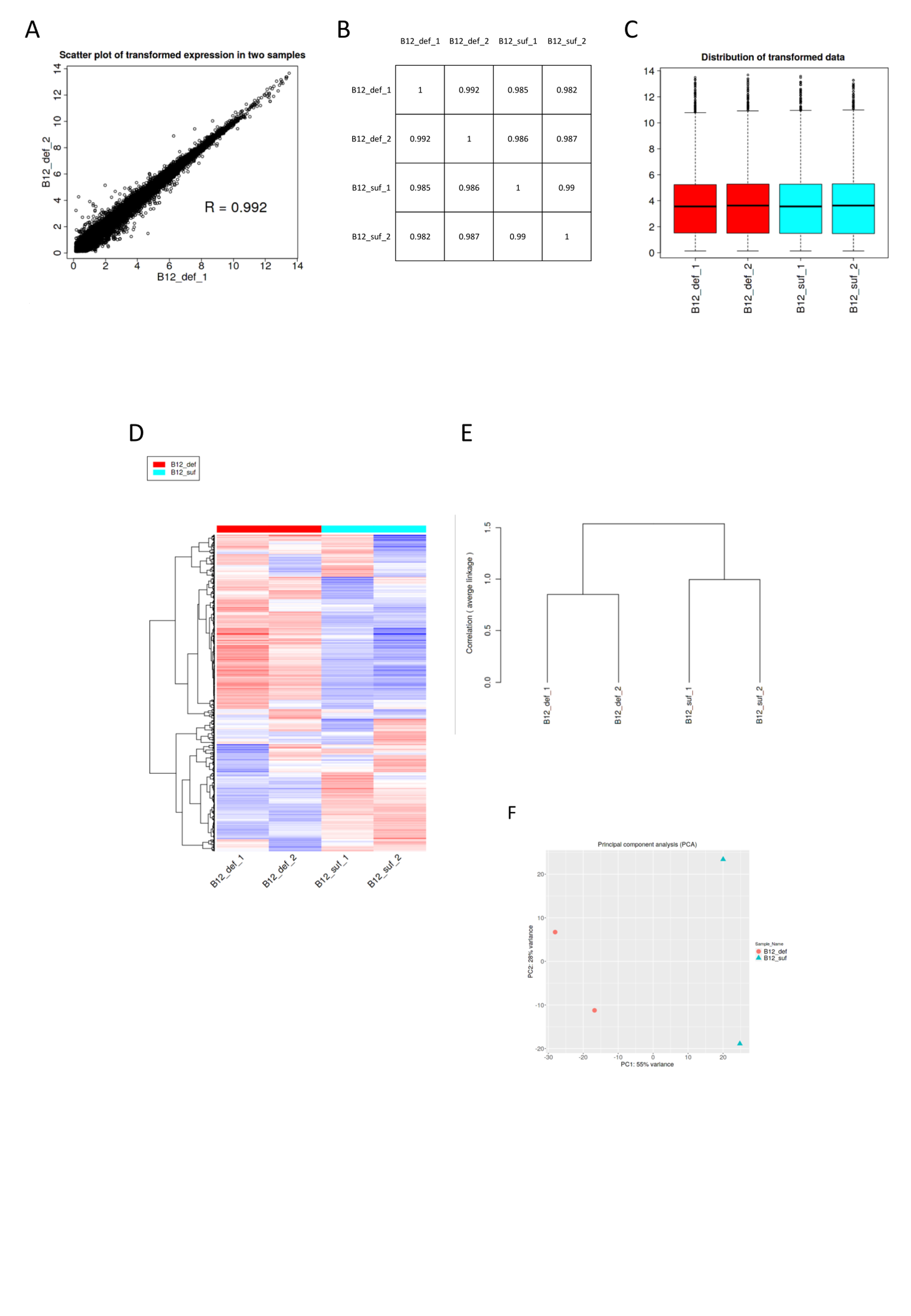

Supplement: Supplementary file 3 — RNA-seq for understanding the effects of vitamin B12 removal on astrocyte culture. (A) Scatter plot of the first two samples. (B) Correlation matrix reporting Pearson’s correlation coefficients. (C) Distribution of transformed data. (D) Heatmap of 1000 most variable genes. (E) Hierarchical clustering tree. (F) PCA analyses. B12_def: astrocytes cultured in free VitB12 medium; B12_suf: astrocytes cultured in normal medium (PNG 432 kb) [file 10142_2023_969_Fig7_ESM.png]

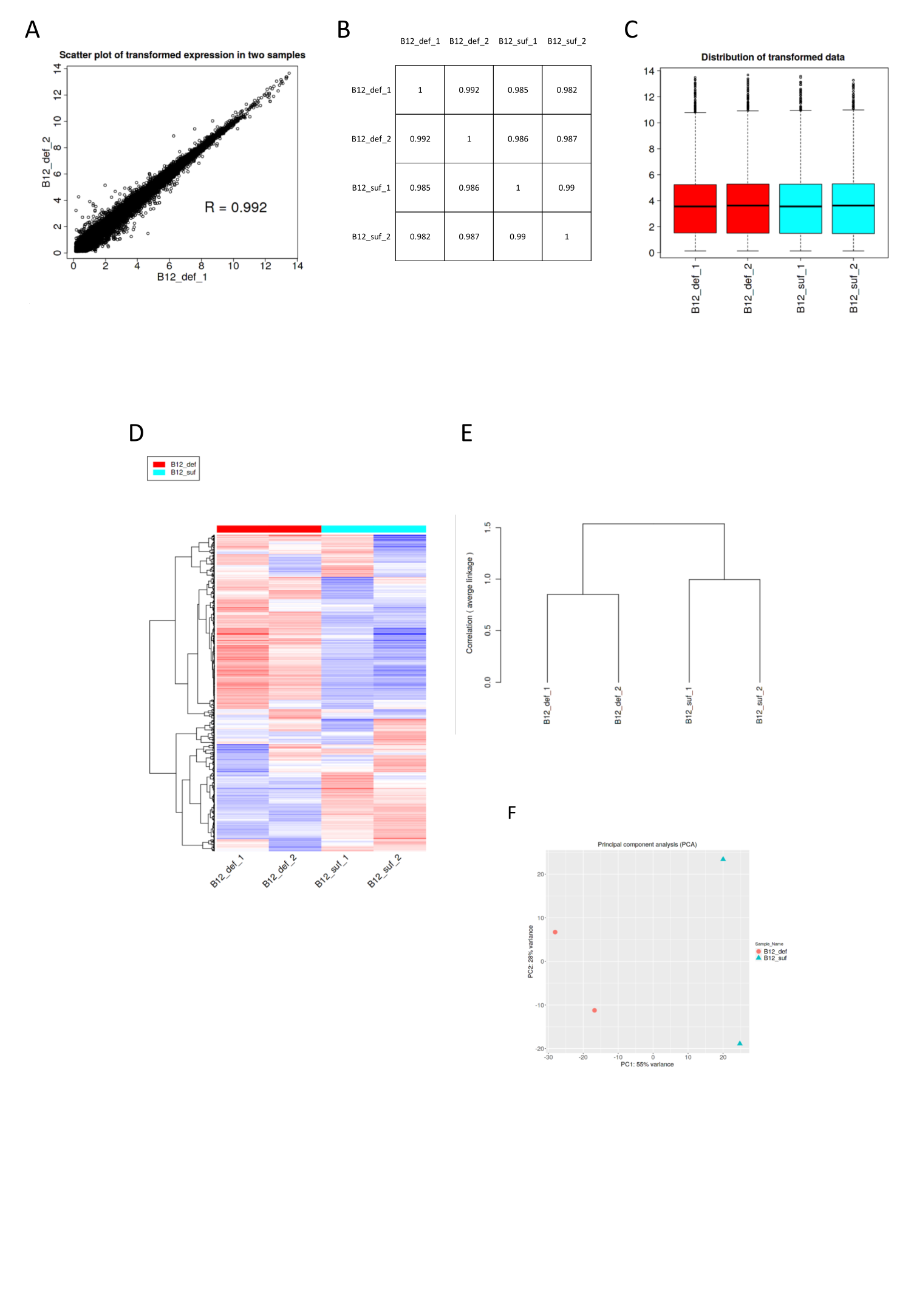

Supplement: Supplementary file 4 — High Resolution Image (TIFF 346 kb) [file 10142_2023_969_MOESM2_ESM.tiff]

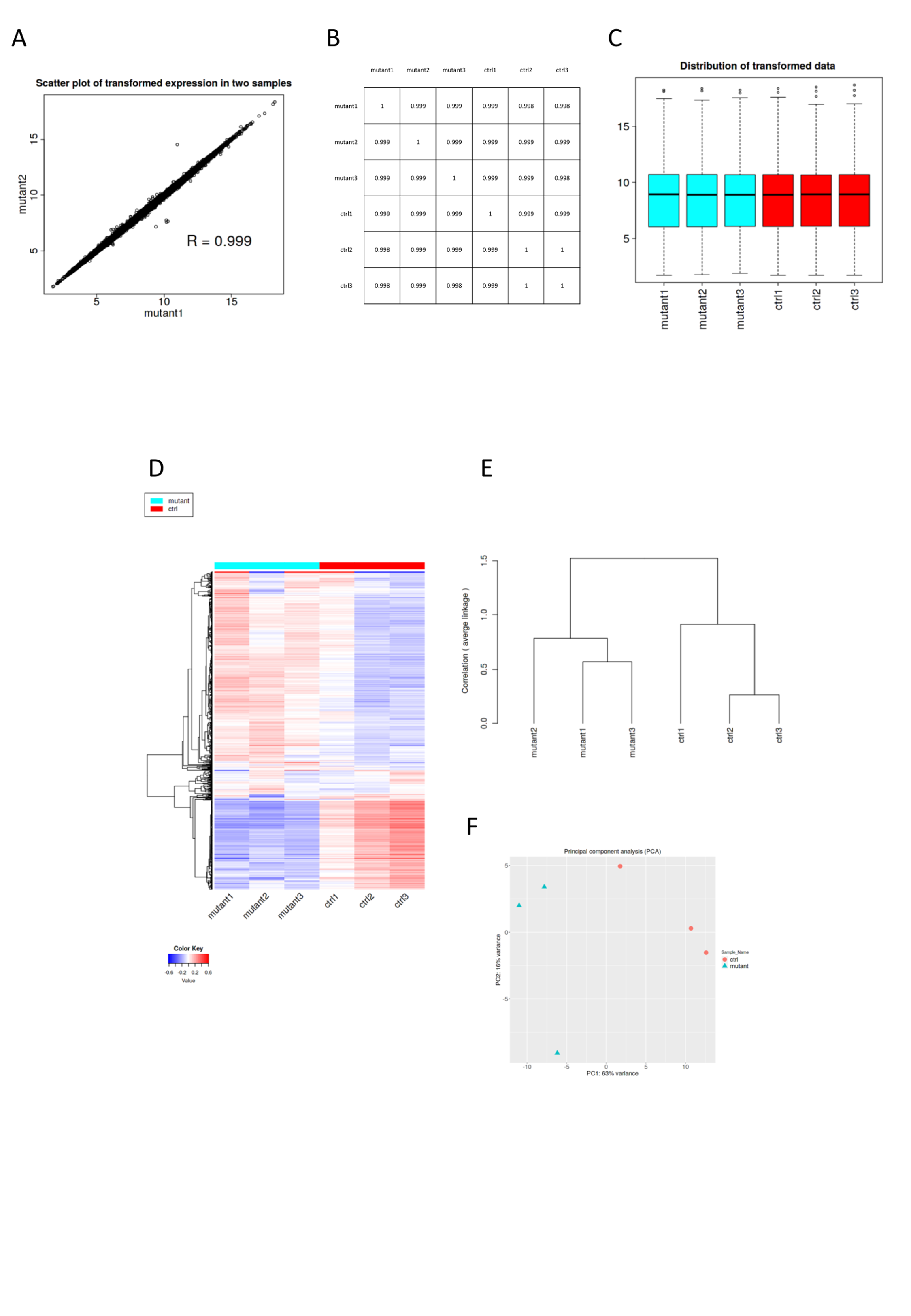

Supplement: Supplementary file 5 — CblX disease is both an inborn error of cobalamin metabolism and a ribosomopathy. (A) Scatter plot of the first two samples. (B) Correlation matrix reporting Pearson’s correlation coefficients. (C) Distribution of transformed data. (D) Heatmap of 1000 most variable genes. (E) Hierarchical clustering tree. (F) PCA analyses. ctrl: wild-type mouse; mutant: mouse carrying THAP11F80L/F80L mutated protein (PNG 418 kb) [file 10142_2023_969_Fig8_ESM.png]

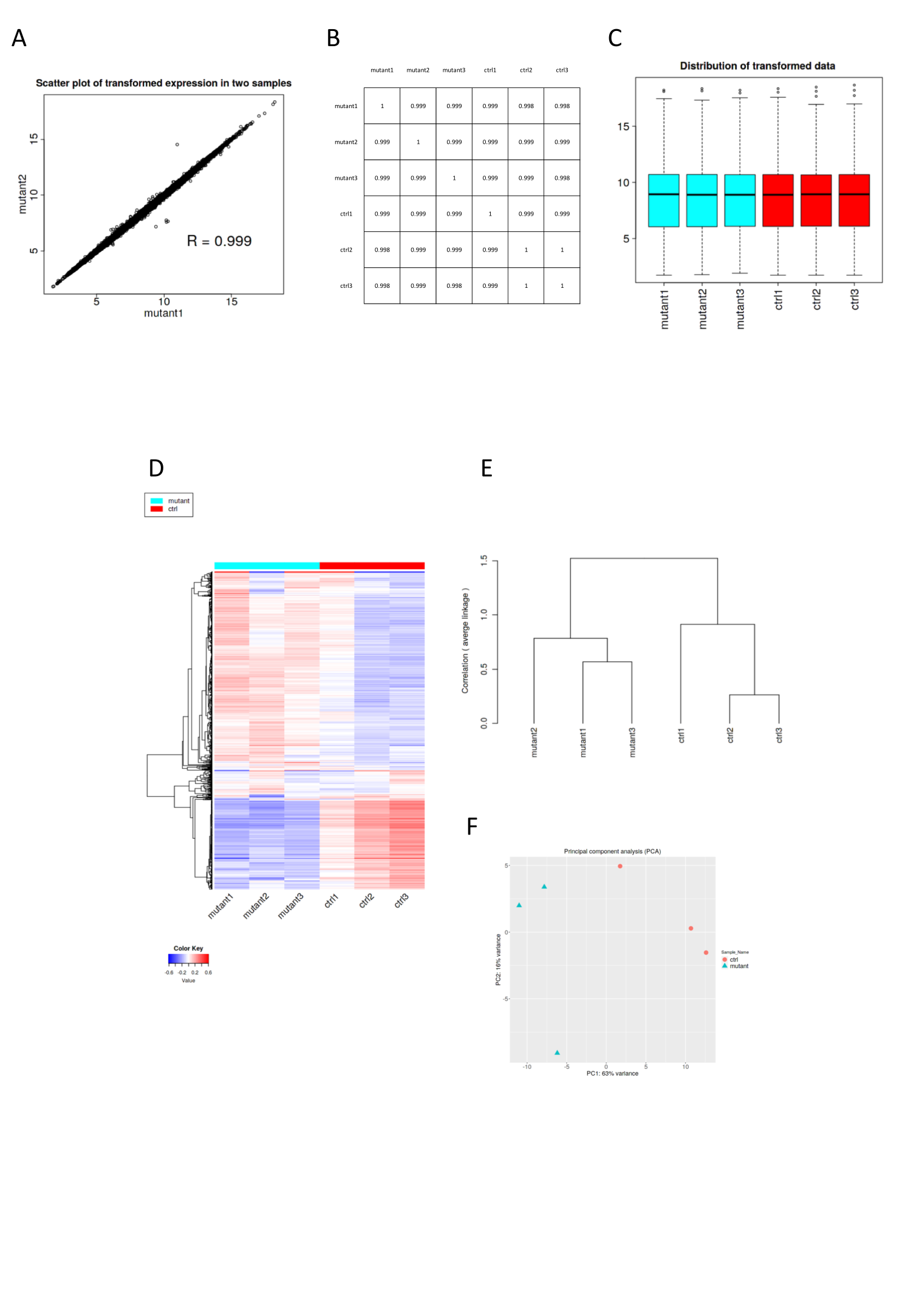

Supplement: Supplementary file 6 — High Resolution Image (TIFF 345 kb) [file 10142_2023_969_MOESM3_ESM.tiff]

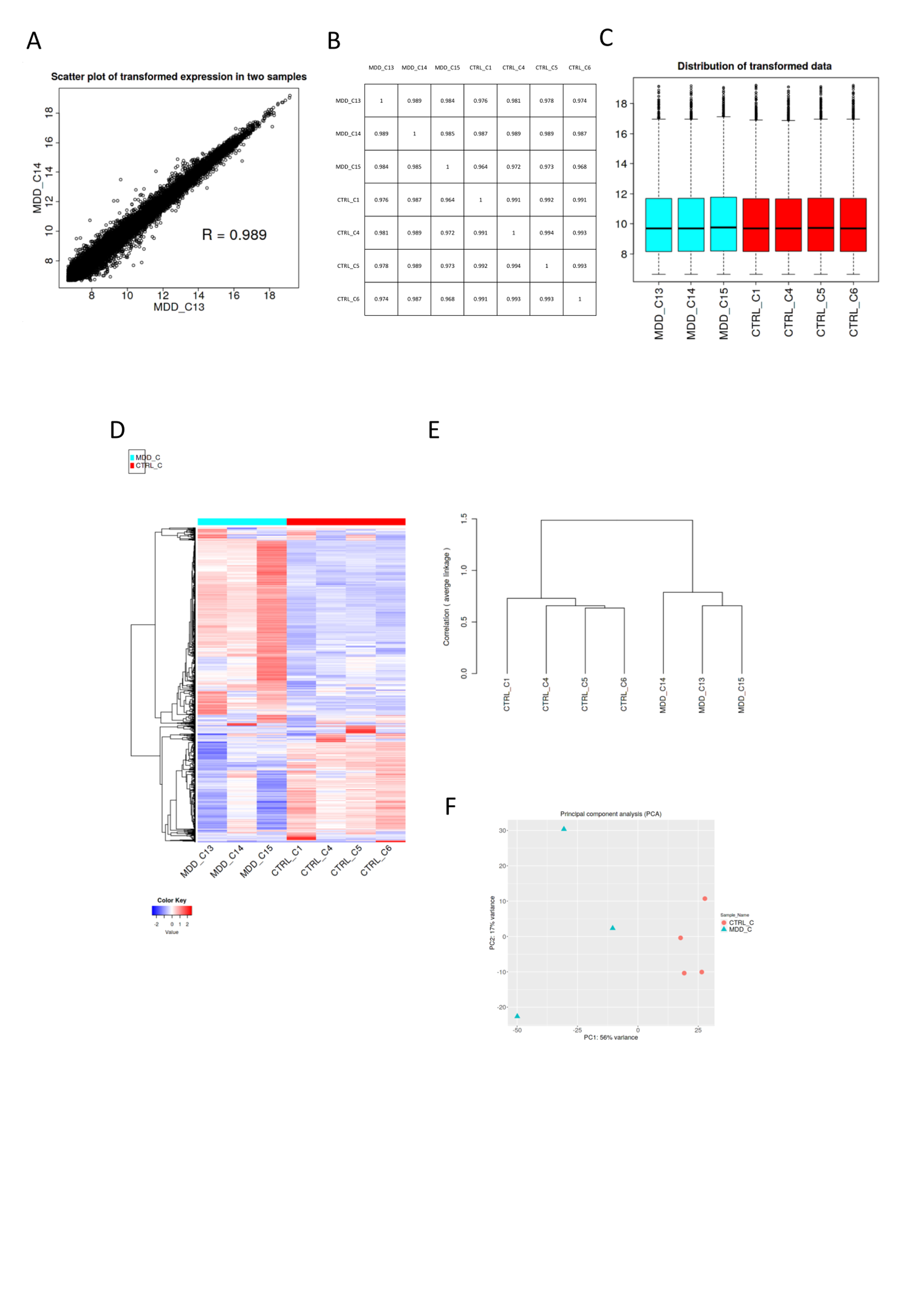

Supplement: Supplementary file 7 — Wnt-signaling pathways are dysregulated in female cerebellum following an early methyl donor deficiency in a rat nutritional model. (A) Scatter plot of the first two samples. (B) Correlation matrix reporting Pearson’s correlation coefficients. (C) Distribution of transformed data. (D) Heatmap of 1000 most variable genes. (E) Hierarchical clustering tree. (F) PCA analyses. CTRL: rats under standard diet; MDD_C: rats under diet deficient in folate (VitB9) and VitB12 and lowered in choline (PNG 543 kb) [file 10142_2023_969_Fig9_ESM.png]

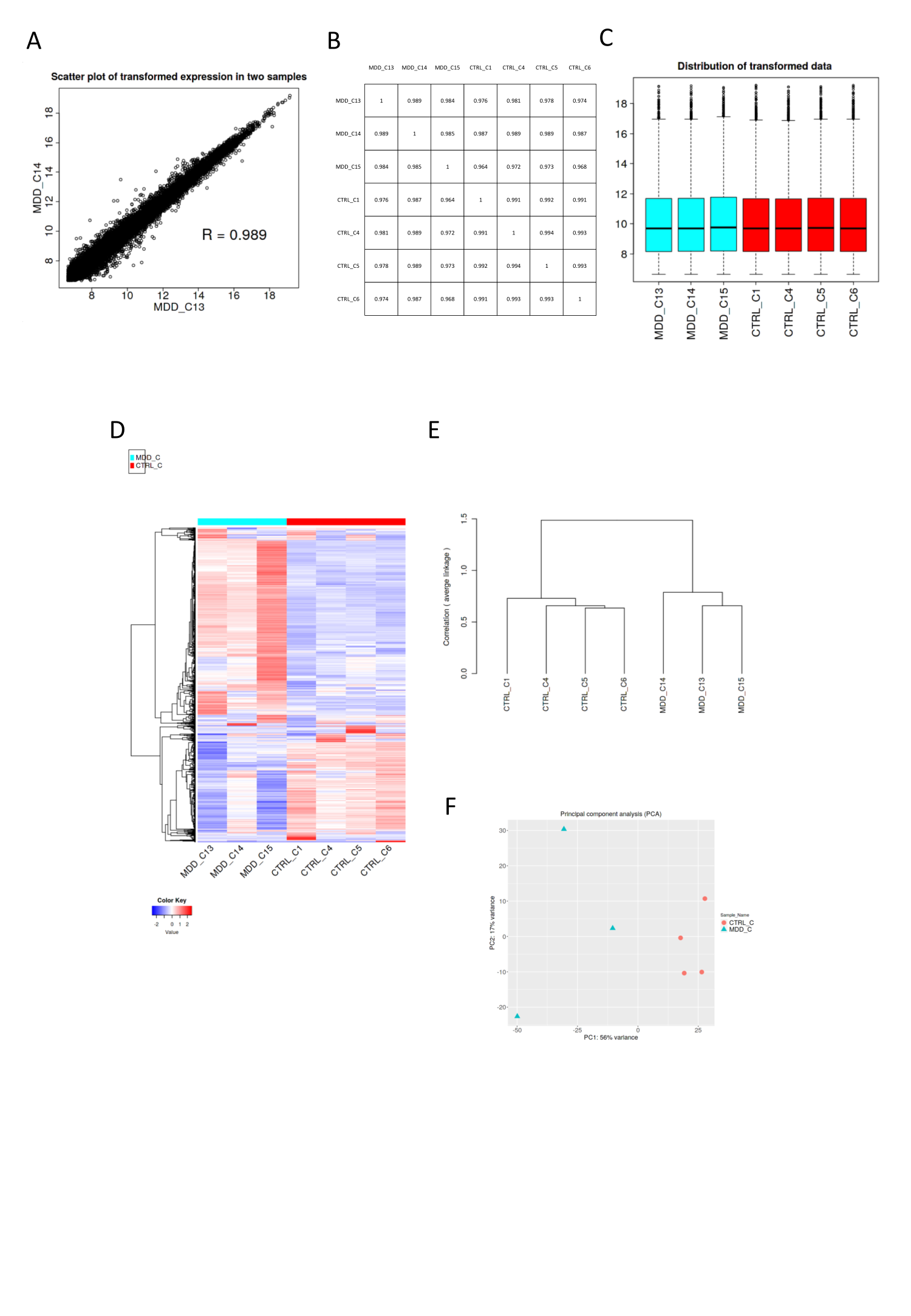

Supplement: Supplementary file 8 — High Resolution Image (TIFF 428 kb) [file 10142_2023_969_MOESM4_ESM.tiff]

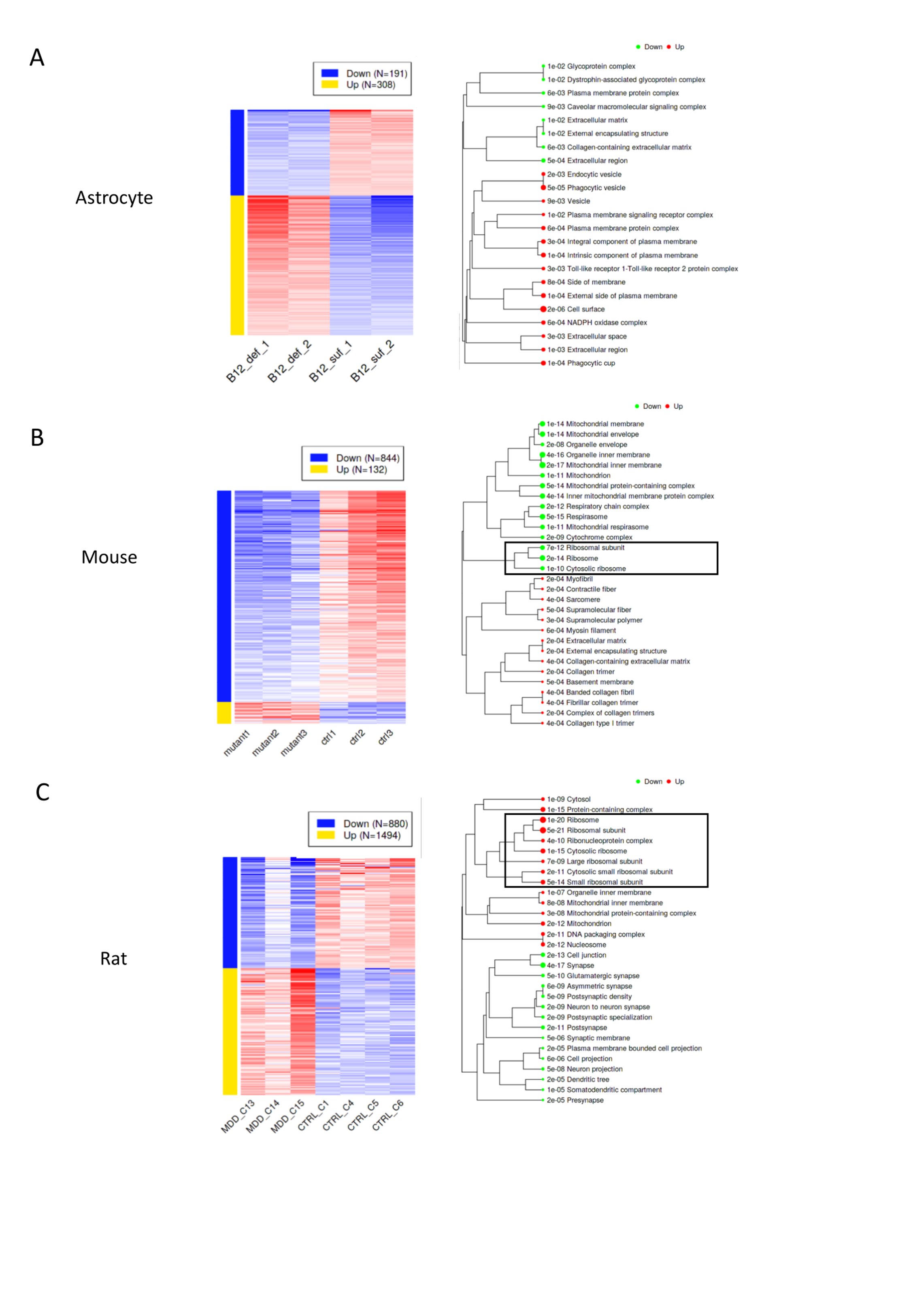

Supplement: Supplementary file 9 — Differentially Expressed Genes analysis (DEG). Heatmap (left panel) and hierarchical clustering tree (right panel) from DEG analysis considering GO Cellular Component gene set from astrocyte (A), mouse (B) and rat (C) models. Green dots in hierarchical clustering tree indicate a downregulated set of genes involved in a specific pathway, while red dots indicate upregulated ones. Numbers before the name of gene dataset is an adjusted p-value. The black square indicates the GO: Cellular component related to ribosomes (PNG 1036 kb) [file 10142_2023_969_Fig10_ESM.png]

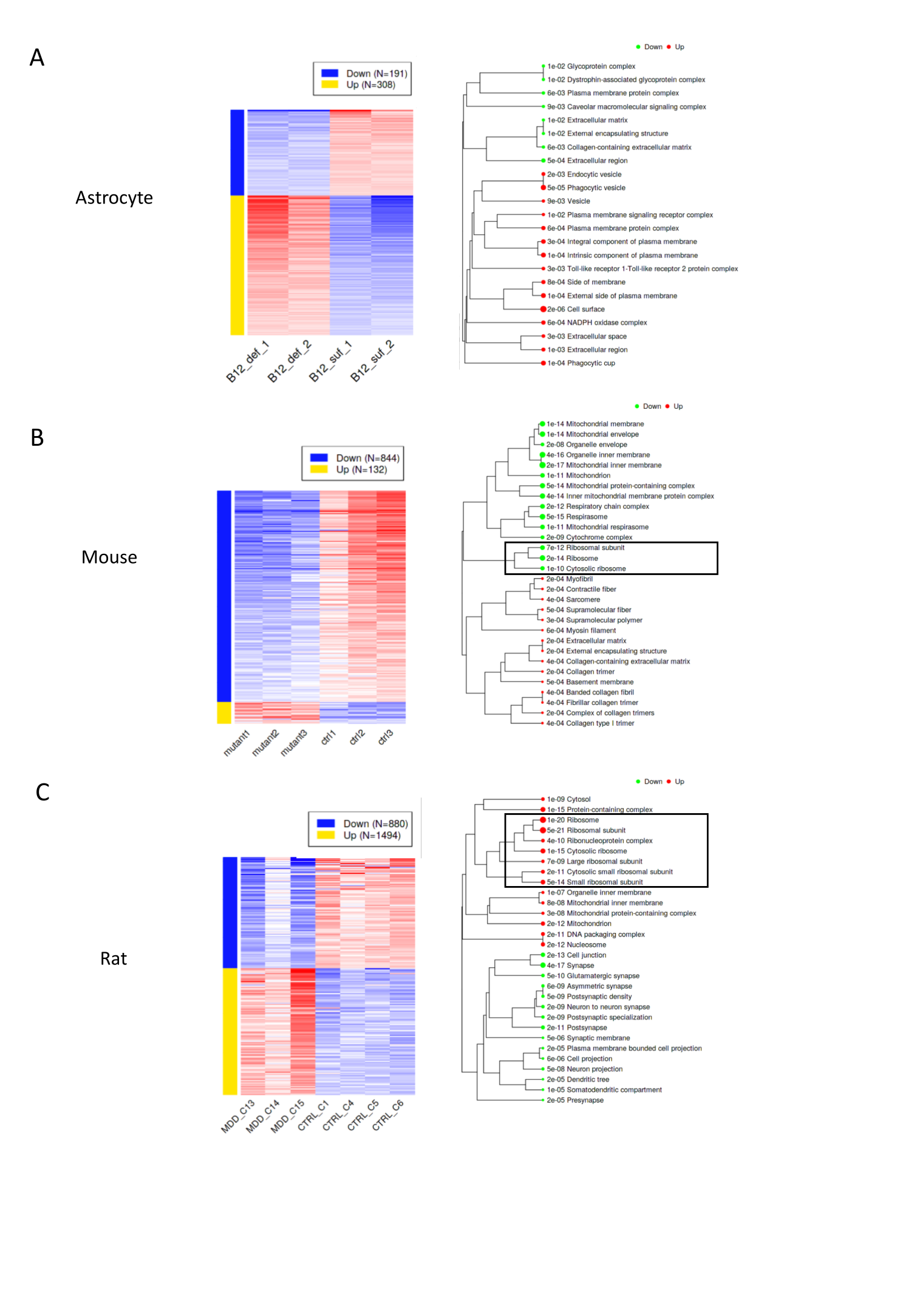

Supplement: Supplementary file 10 — High Resolution Image (TIFF 820 kb) [file 10142_2023_969_MOESM5_ESM.tiff]

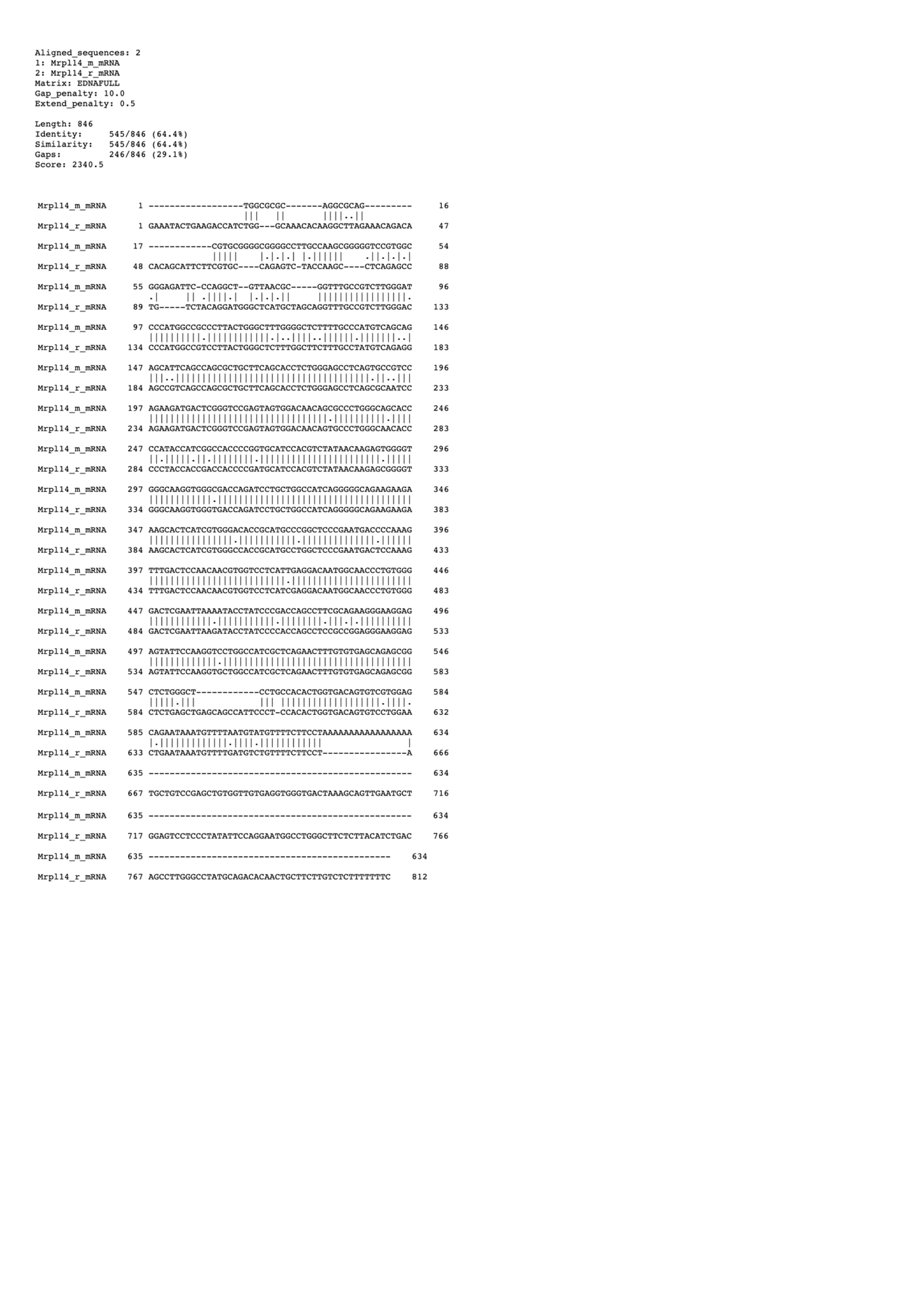

Supplement: Supplementary file 11 — Pairwise Sequence Alignment of Mrpl14 mRNA from rat and mouse (PNG 446 kb) [file 10142_2023_969_Fig11_ESM.png]

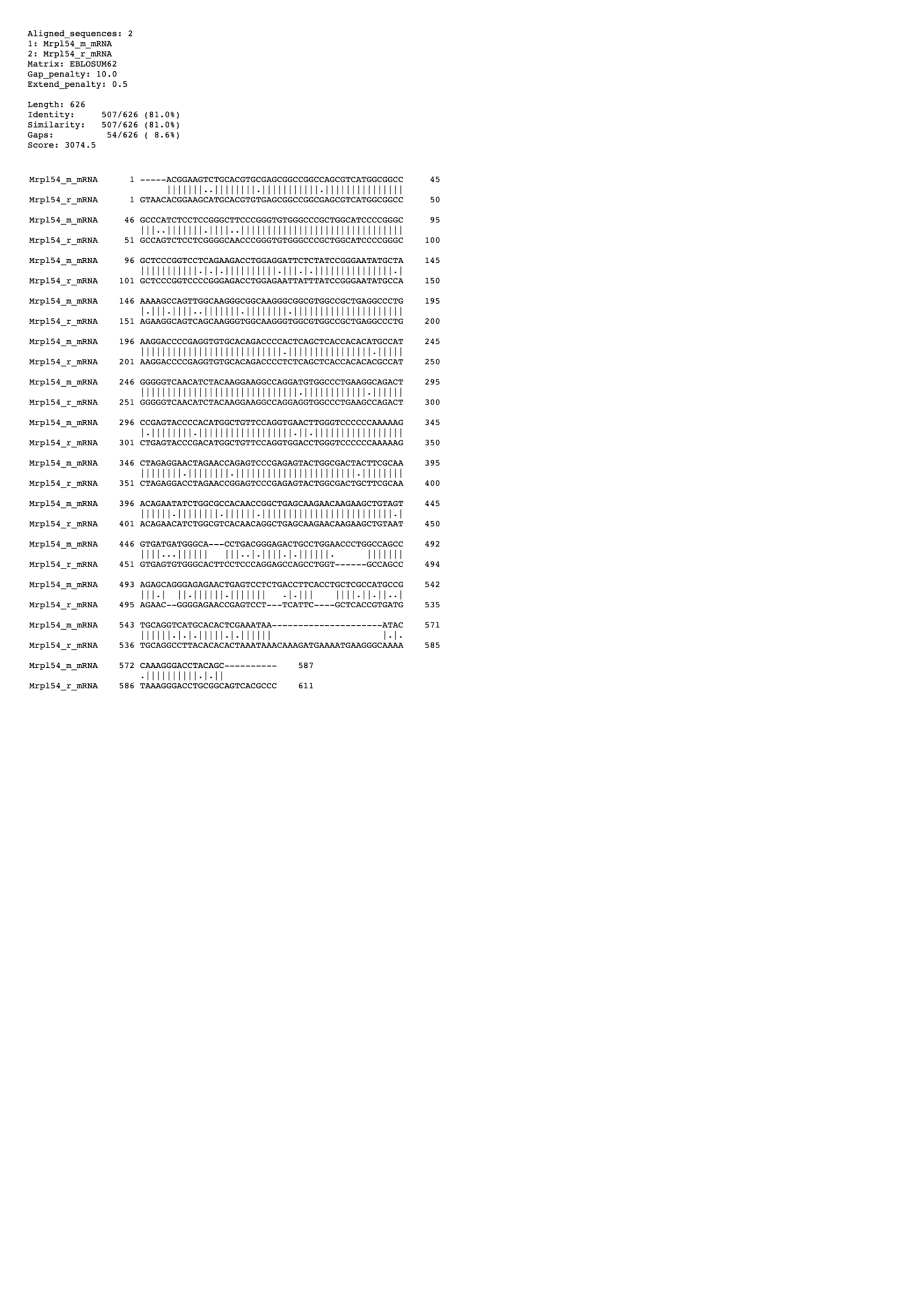

Supplement: Supplementary file 13 — Pairwise Sequence Alignment of Mrpl54 mRNA from rat and mouse (PNG 367 kb) [file 10142_2023_969_Fig12_ESM.png]

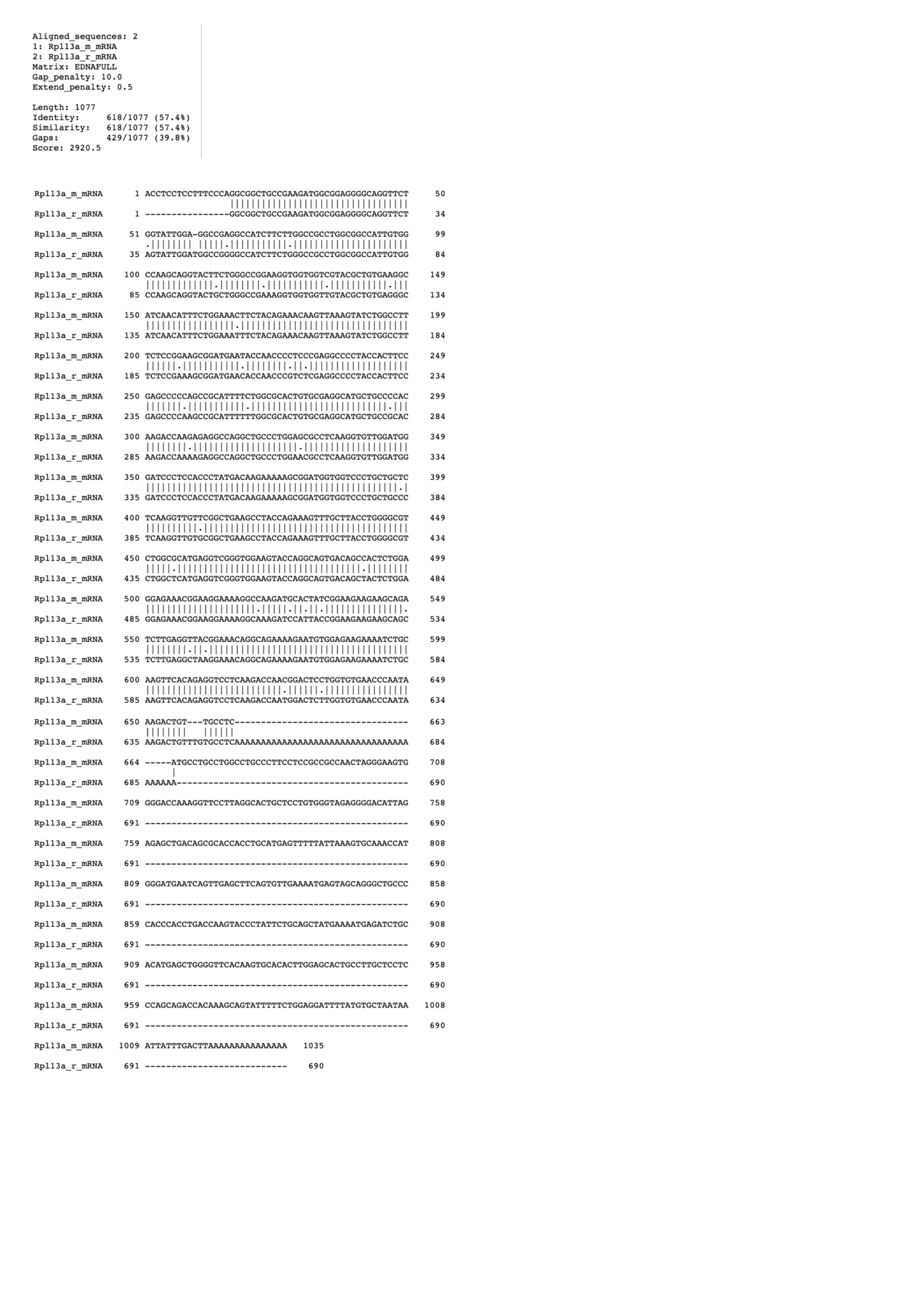

Supplement: Supplementary file 15 — Pairwise Sequence Alignment of Rpl11a mRNA from rat and mouse (PNG 539 kb) [file 10142_2023_969_Fig13_ESM.png]

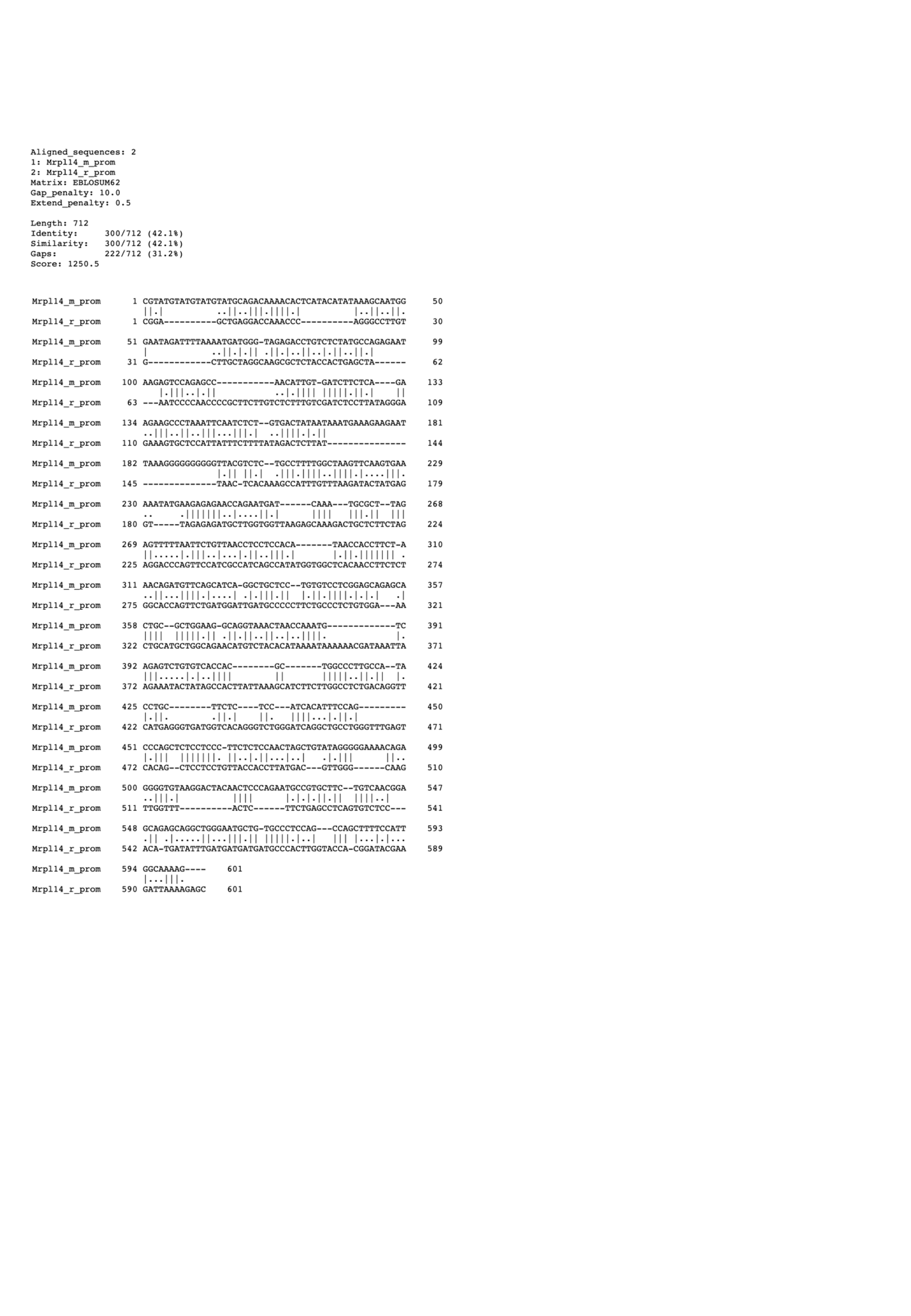

Supplement: Supplementary file 17 — Pairwise Sequence Alignment of Mrpl14 promoter sequence from rat and mouse (PNG 387 kb) [file 10142_2023_969_Fig14_ESM.png]

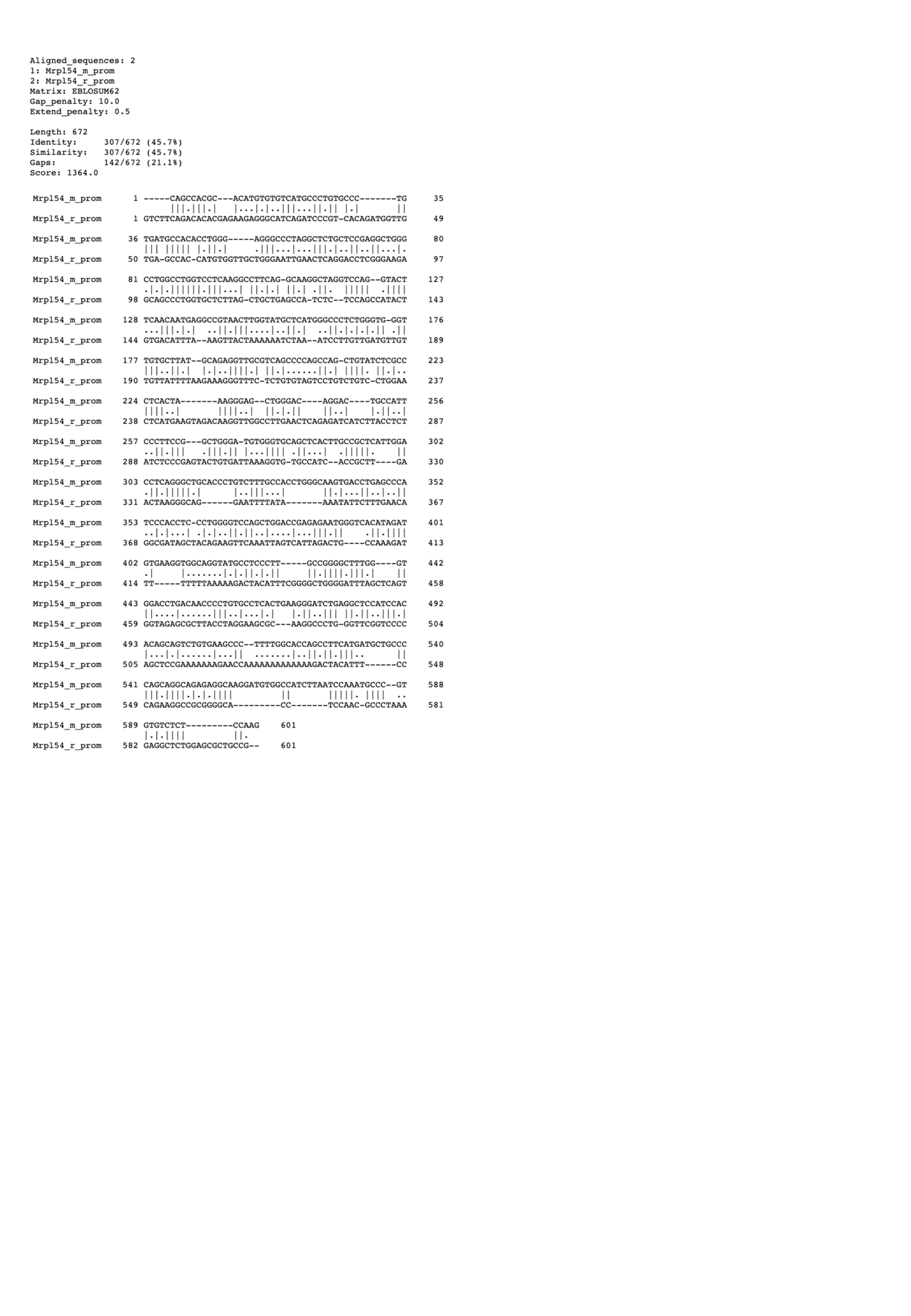

Supplement: Supplementary file 19 — Pairwise Sequence Alignment of Mrpl54 promoter sequence from rat and mouse (PNG 379 kb) [file 10142_2023_969_Fig15_ESM.png]

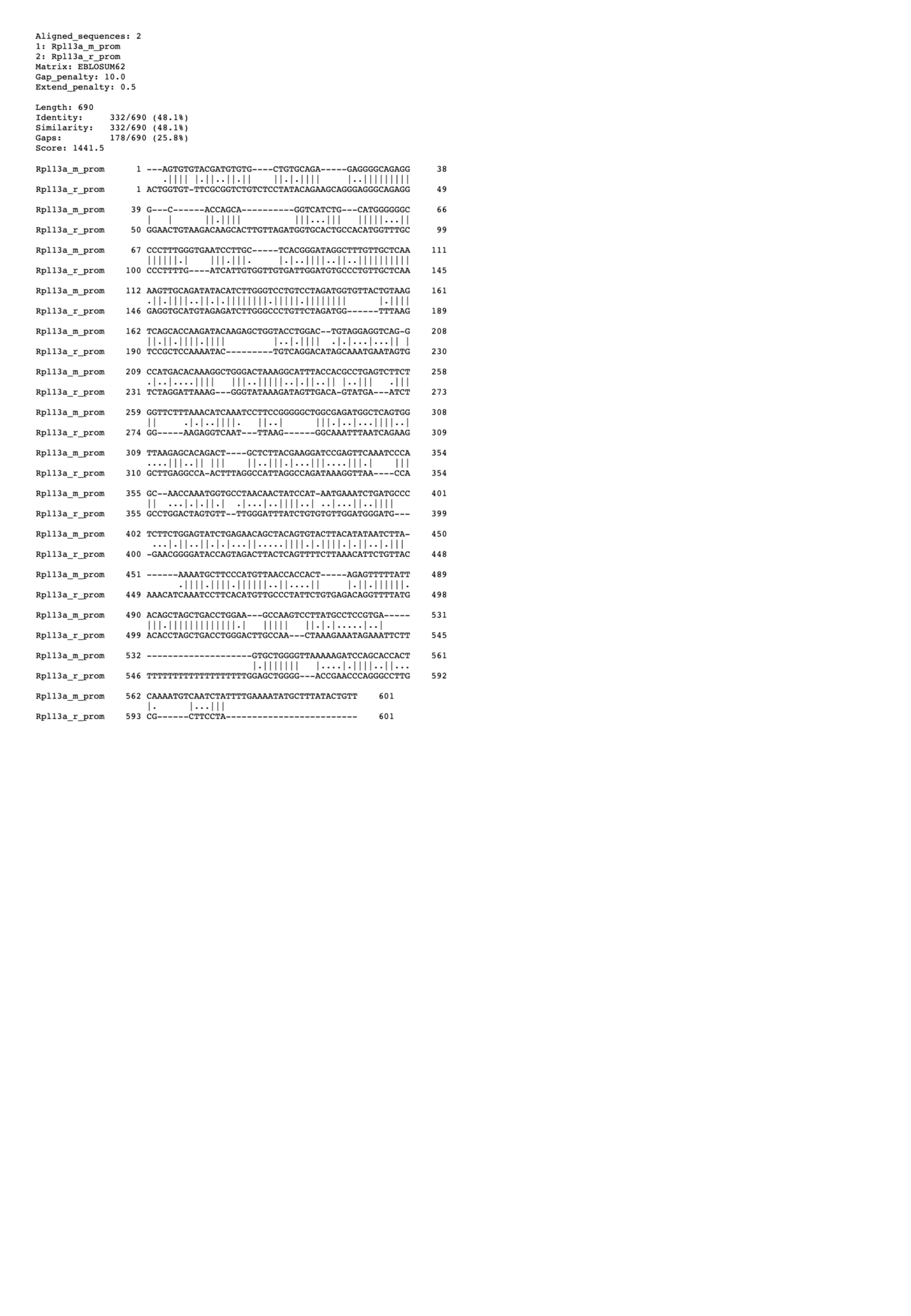

Supplement: Supplementary file 21 — Pairwise Sequence Alignment of Rpl13a promoter sequence from rat and mouse (PNG 379 kb) [file 10142_2023_969_Fig16_ESM.png]

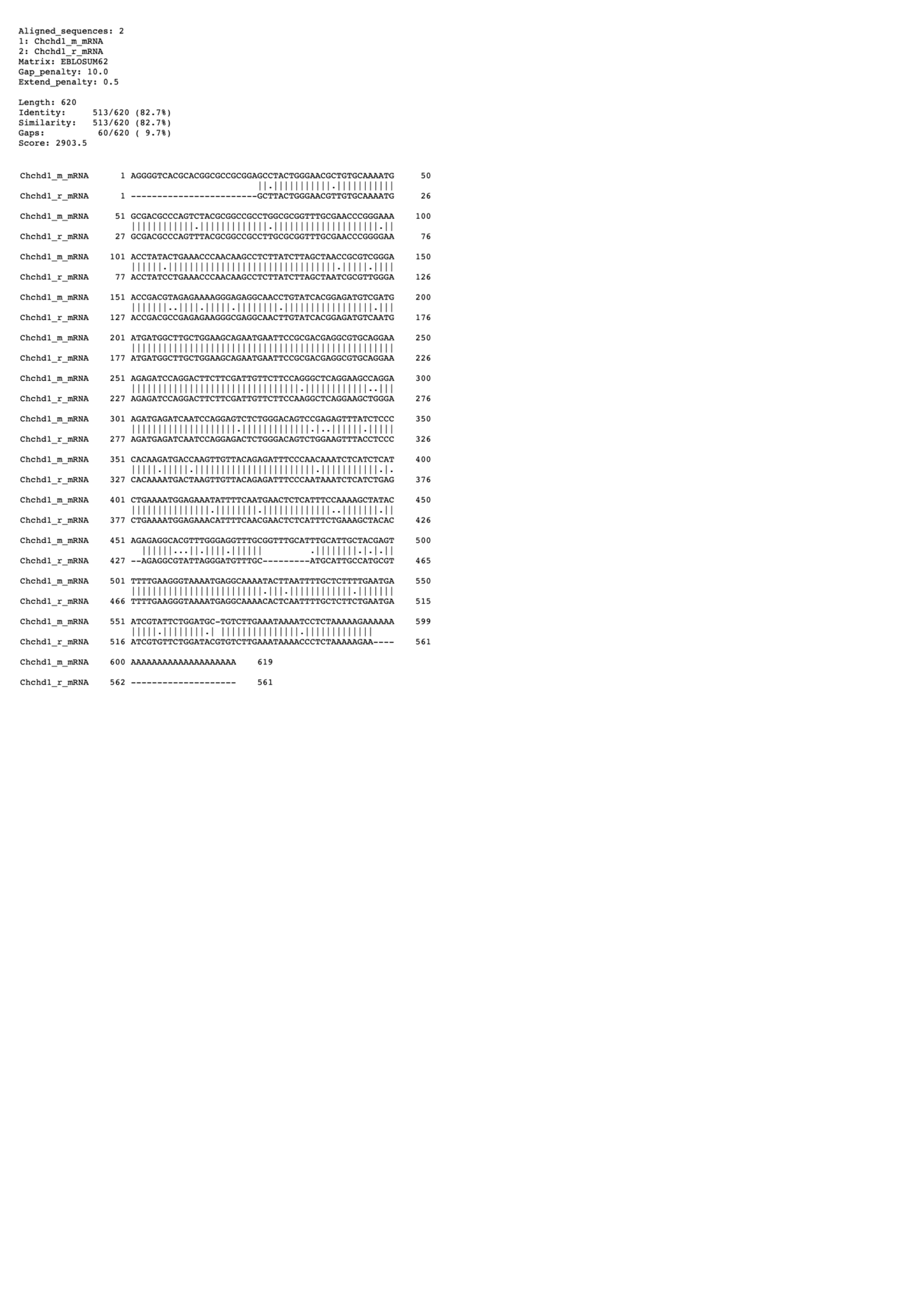

Supplement: Supplementary file 23 — Pairwise Sequence Alignment of Chchd1 mRNA from rat and mouse (PNG 363 kb) [file 10142_2023_969_Fig17_ESM.png]

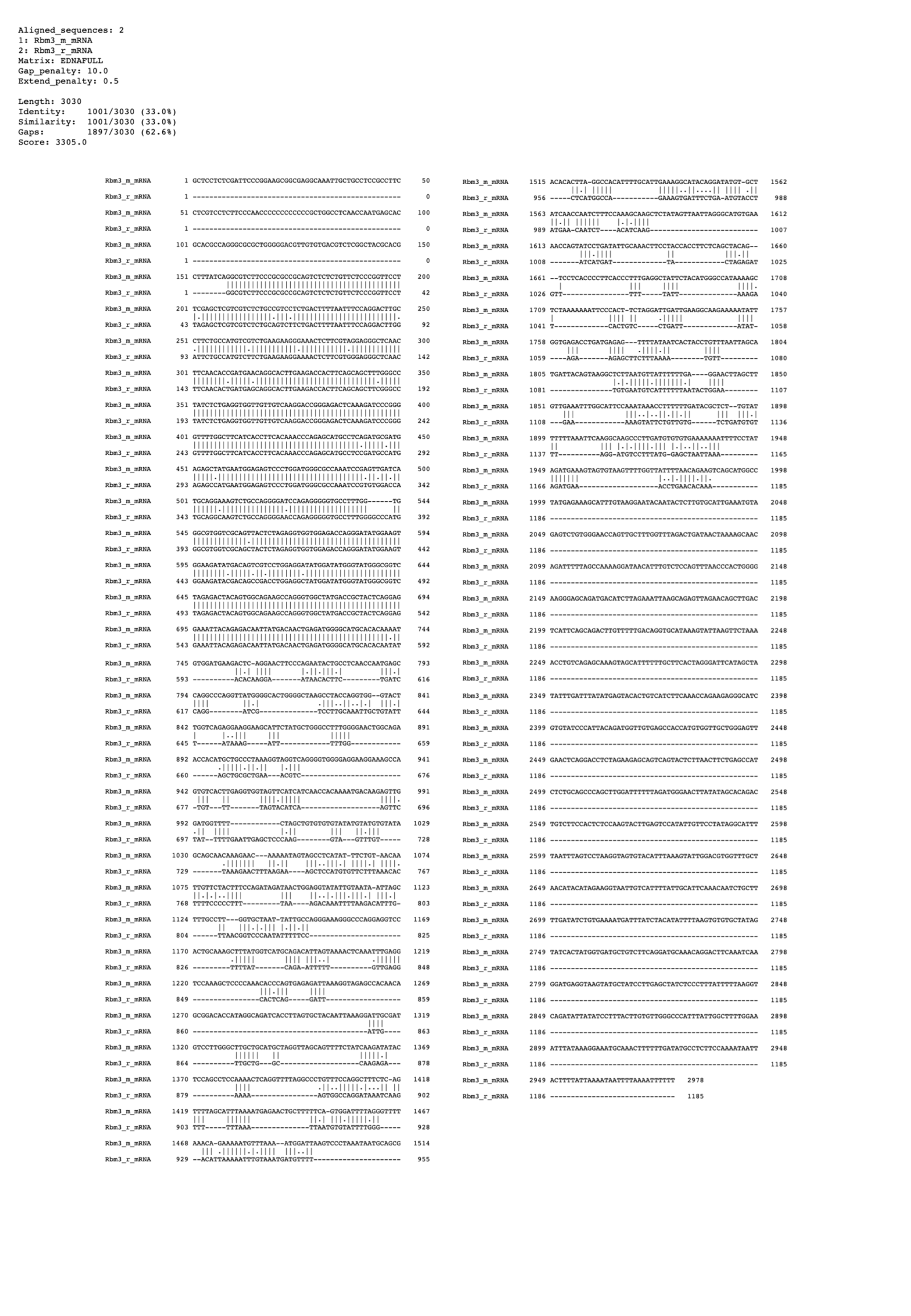

Supplement: Supplementary file 25 — Pairwise Sequence Alignment of Rbm3 mRNA from rat and mouse (PNG 927 kb) [file 10142_2023_969_Fig18_ESM.png]

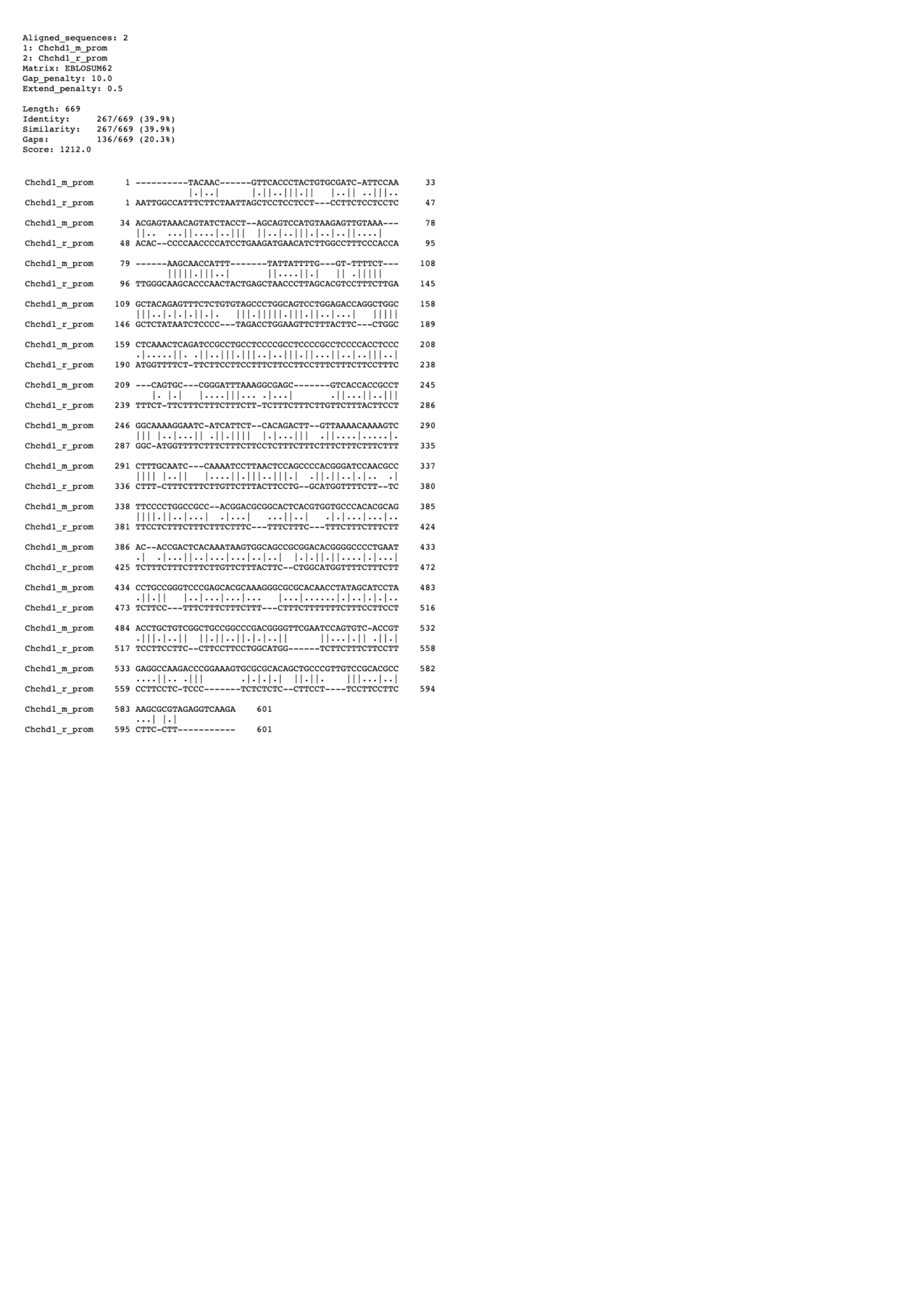

Supplement: Supplementary file 27 — Pairwise Sequence Alignment of Chchd1 promoter sequence from rat and mouse (PNG 370 kb) [file 10142_2023_969_Fig19_ESM.png]

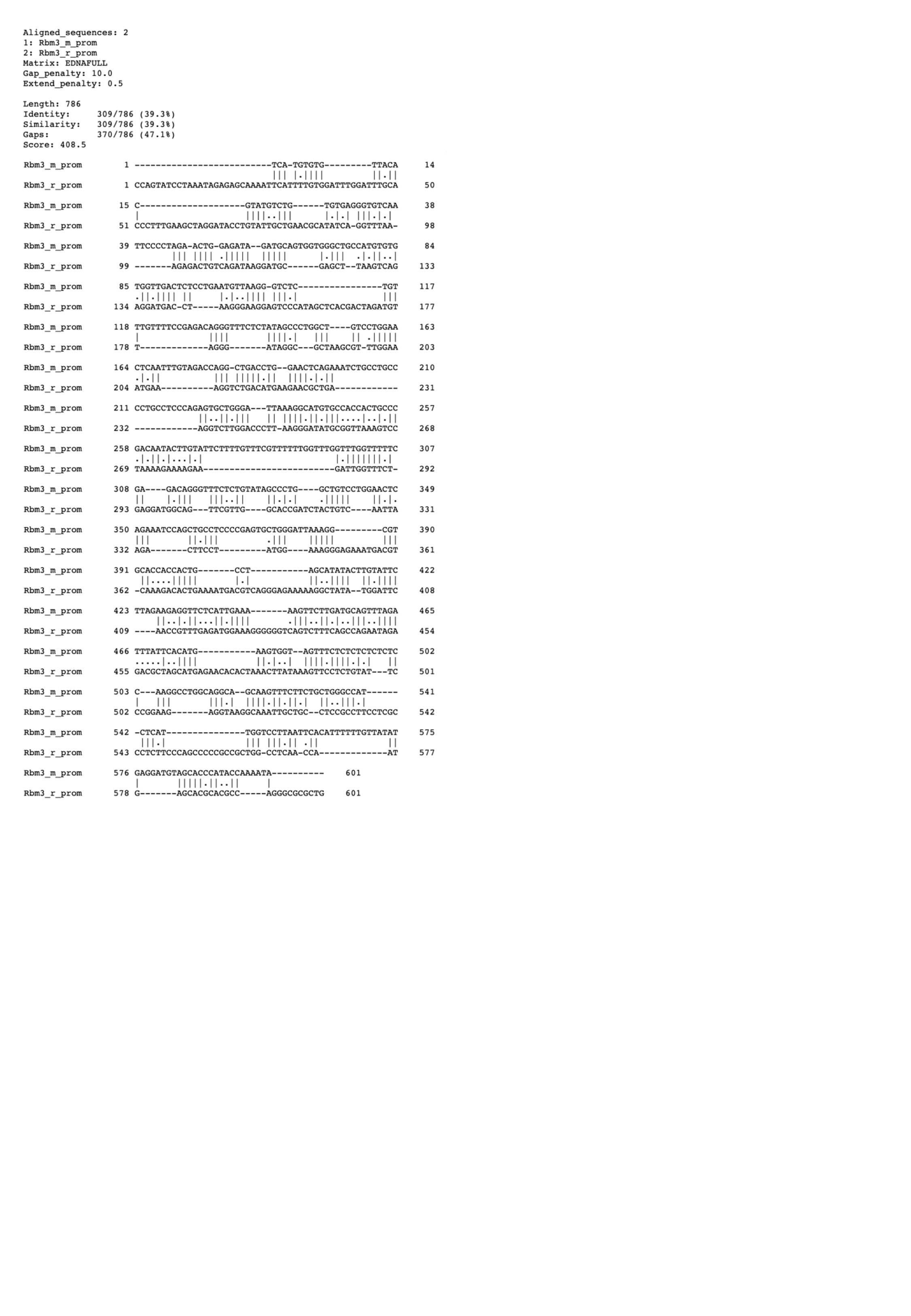

Supplement: Supplementary file 29 — Pairwise Sequence Alignment of Rbm3 promoter sequence from rat and mouse (PNG 390 kb) [file 10142_2023_969_Fig20_ESM.png]
